# Southward impact excavated magma ocean at the lunar South Pole–Aitken basin

**DOI:** 10.1038/s41586-025-09582-y

**Published:** 2025-10-08

**Authors:** Jeffrey C. Andrews-Hanna, William F. Bottke, Adrien Broquet, Alexander J. Evans, Gabriel Gowman, Brandon C. Johnson, James T. Keane, Janette N. Levin, Ananya Mallik, Simone Marchi, Samantha A. Moruzzi, Arkadeep Roy, Shigeru Wakita

**Affiliations:** 1 Lunar and Planetary Laboratory, University of Arizona, Tucson, AZ USA; 2 Center for Lunar Origin and Evolution, NASA Solar System Exploration Research Virtual Institute (SSERVI), Boulder, CO USA; 3 Southwest Research Institute, Boulder, CO USA; 4 Institute of Space Research, German Aerospace Center (DLR), Berlin, Germany; 5 Department of Earth, Environmental, and Planetary Sciences, Brown University, Providence, RI USA; 6 Department of Earth, Atmospheric, and Planetary Sciences, Purdue University, West Lafayette, IN USA; 7 Department of Physics and Astronomy, Purdue University, West Lafayette, IN USA; 8 Jet Propulsion Laboratory, California Institute of Technology, Pasadena, CA USA; 9 Department of Geosciences, University of Arizona, Tucson, AZ USA

**Keywords:** Inner planets, Geodynamics, Inner planets

## Abstract

The ancient South Pole–Aitken impact basin provides a key data point for our understanding of the evolution of the Moon, as it formed during the earliest pre-Nectarian epoch of lunar history^1^, excavated more deeply than any other known impact basin^2,3^ and is found on the lunar far side, about which less is known than the well-explored near side. Here we show that the tapering of the basin outline and the more gradual topographic and crustal thickness transition towards the south support a southward impact trajectory, opposite of that commonly assumed. A broad thorium-rich and iron-rich ejecta deposit southwest of the basin is consistent with partial excavation of late-stage magma ocean liquids. These observations indicate that thorium-rich magma ocean liquids persisted only beneath the southwestern half of the basin at the time of impact, matching predictions for the transition from a global magma ocean to a local enrichment of potassium, rare-earth elements and phosphorus (KREEP) in the near-side Procellarum KREEP Terrane. These results have important implications for the upcoming human exploration of the lunar south pole by Artemis, as proposed landing sites are now recognized to sit on the downrange rim and thorium-rich impact ejecta of the basin.

## Abstract (web summary)

Observations of the shape, topography, crustal thickness and surface composition of the South Pole–Aitken impact basin on the Moon suggest a southward impact trajectory and the excavation of a discontinuous remnant magma ocean from beneath the crust.

## Main

The South Pole–Aitken (SPA) basin-forming impact occurred during a critical stage in lunar evolution during which the Moon was subjected to a heavy bombardment of impacts^4^ while potentially still in the final stages of magma ocean crystallization^5,6^. The Moon experienced a global magma ocean after its accretion from an impact-generated disk^7^. The crystallization of this magma ocean would have generated a cumulate mantle with an inverted density profile, culminating in the formation of dense, titanium-rich, ilmenite (FeTiO_3_)-bearing cumulates and a final liquid strongly enriched in incompatible elements such as potassium, rare-earth elements and phosphorus (KREEP)^8^, including the element thorium (Th). Although the precise details remain uncertain, the final outcome of magma ocean crystallization and the associated overturn of the cumulate mantle was the concentration of both ilmenite-bearing cumulates and KREEP on the near side in the Procellarum KREEP Terrane (PKT) by approximately 4.34–4.37 Ga (refs. ^9,10^). The composition of the SPA basin floor suggests a mantle-excavating impact before the overturn of the buoyantly unstable post-magma ocean cumulates^11,12^, whereas increased concentrations of titanium and thorium within the basin^13^ suggest that the impact excavated into an ilmenite-rich and KREEP-rich reservoir^11^. The age of the SPA basin has been estimated at 4.25 Ga based on a near-side sample^14^ or 4.33 Ga based on a lunar meteorite^15^, whereas crater retention model ages of 4.31 Ga (ref. ^16^) or 4.33–4.39 Ga (ref. ^17^) depend on the uncertain early bombardment history. Thus, important questions remain about the age of the basin, the timing and nature of magma ocean crystallization and the relation between these two pivotal events in early lunar evolution.

## Basin shape and impact trajectory

The SPA basin is quasi-elliptical in shape^18,19^, as is typical for giant impact basins^20^. The approximately north–south orientation of the long axis of the basin, as well as the volume equivalence of the crustal deficit within the basin with the crustal excess of the far-side highlands, suggested that the ejecta from a northward-directed impact may contribute to the near side–far side asymmetry in topography and crustal thickness^21^. If correct, this scenario would predict little or no ejecta at the southern end of the basin^3^. However, the long axis of the basin is oriented obliquely to the peak crustal thickening of the far-side highlands^18^, predicted ejecta patterns do not match the far-side crustal excess^22^ and similar downrange excesses in crustal thickness or topography of comparable magnitude are not observed around other giant basins of comparable size, including Hellas and Utopia on Mars and Sputnik on Pluto (see Supplementary Discussion). Thus, it seems that the lunar topographic and crustal thickness asymmetry is dominantly a result of other processes (see review in ref. ^23^). A northward impact trajectory has also been suggested on the basis of magnetic anomalies in the northern portion of the SPA basin interpreted as magnetized impactor material^24^, although anomalies of comparable scale and magnitude are found elsewhere in the far-side highlands further from the basin and alternative explanations exist^25,26^. Although the impact direction of the SPA basin is critical for understanding the distribution and composition of its ejecta, there has been little consideration of the opposite southward impact trajectory^27^ and the implications for the upcoming exploration of the southern rim of the basin.

Another constraint on the direction of projectile motion is the shape of the resulting basin. Although most giant impact basins are elongated as a result of the effect of planetary curvature on basin formation^20^, each of these elongated basins also exhibits a pronounced tapering, suggesting that this tapering is a fundamental aspect of basin structure and formation. This tapering is in the downrange direction for those basins with independent evidence of the direction of projectile motion as discussed below. Impact basins form as a transient cavity of excavated and displaced material expands outward from the footprint of the projectile on the surface. This expansion is a function of impact velocity and angle (*θ*), with the final crater diameter scaling approximately as sin(*θ*)^1/3^, such that more oblique impacts generate proportionally smaller basins^28,29^. For basins that are an appreciable fraction of the planetary radius in size, the angle between the projectile motion and the surface is more oblique on the downrange end of the basin owing to the curvature of the surface, which should lead to a cross-track narrowing of the basin at that end. At larger sizes and more oblique impacts, projectile decapitation is increasingly likely, in which a portion of the projectile continues on a path that does not intersect with the planetary surface, leading to enhanced excavation or scouring of the surface in the downrange direction and generating distinctive crater and basin shapes^30^.

In this work, we first compare the SPA basin to other giant impact basins for which independent evidence of the projectile motion exists (see Methods). Basin outlines are traced using a combination of Bouguer gravity, gravity gradients^31^ and topography. Basin tapering is evaluated by fitting the basin outlines to a tapered ellipse, from which we evaluate the tapering *f*, calculated by taking the difference between the basin width at the points midway between the basin centre and the uprange/downrange ends and dividing by the along-track distance between these points. We also use a geometrical model of basin elongation and tapering based on a simple representation of the physics of basin excavation, taking into account the variation of the local impact angle with position in the basin (modified from ref. ^20^; see Supplementary Discussion).

**Fig. 1:**
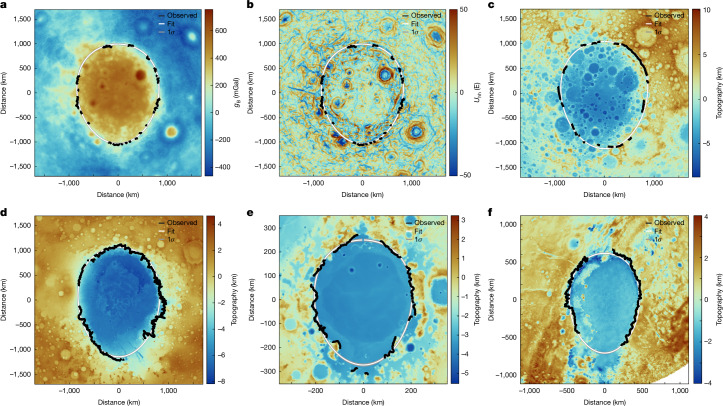
Gravity and topography maps of giant tapered impact basins. **a**–**f**, Maps of the SPA basin in Bouguer gravity (**a**), gravity gradients (**b**) and topography (**c**) in comparison with topographic maps of the Hellas basin on Mars (**d**), the Crisium basin on the Moon (**e**) and the Sputnik basin on Pluto (**f**). All figures are in a basin-centred polar projection with basins oriented with the inferred downrange direction to the bottom. The rim picks are traced in black, with the mean and 1*σ* fits for a tapered ellipse in white and grey. The tracing in **a** and **b** are the same, based on a combination of the two datasets. Source data

Impact basins for which the projectile direction is established from geologic evidence demonstrate that the rim tapers in the downrange direction (Fig. 1). The Hellas basin on Mars was formed by an impact on an eastward trajectory, as supported by ejecta blocks in the Hellas Montes east of the basin^32,33^, and the basin tapers in that direction in topography and gravity (*f* = 0.15 ± 0.01 and *f* = 0.09 ± 0.03, respectively). The Crisium basin on the Moon tapers towards the east in topography (*f* = 0.15 ± 0.02) and gravity (*f* = 0.21 ± 0.02), consistent with the eastward trajectory of the impact as indicated by the ejecta distribution and downrange gap in the basin rings^1,34^. Prominent tapering is also observed in the Smythii basin on the Moon and the Utopia basin on Mars (see Methods), but the directions of projectile motion are not independently known for these basins. The Sputnik basin on Pluto tapers to the south (*f* = 0.11 ± 0.01), matching the southward impact trajectory indicated by the downrange tail as expected to result from projectile decapitation^35^. The SPA basin tapers towards the south in both gravity (*f* = 0.16 ± 0.01) and topography (*f* = 0.18 ± 0.02), supporting a southward impact trajectory, whereas a northward taper and projectile motion direction can be ruled out at the 2*σ* level. Alternative basin outline tracings yield similar results (see Methods). Three-dimensional numerical models of SPA basin formation by oblique impact also support tapering in the downrange direction^24,36,37^.

**Fig. 2:**
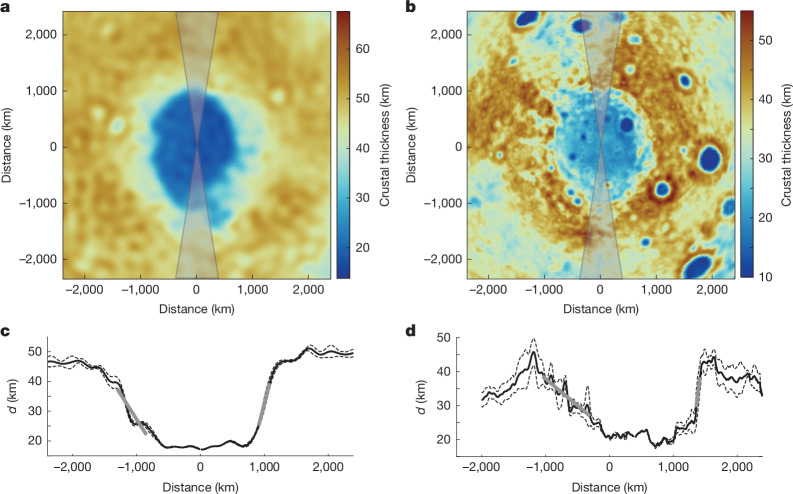
Comparison of the crustal thickness around the Hellas and SPA basins. **a**–**d**, Hellas (**a**,**c**) and SPA (**b**,**d**) basins are shown in map view (**a**,**b**) and profile (**c**,**d**). Profiles were averaged over 15° arcs centred on the long axes of the basins and show more gradual average profiles (grey lines in **c**,**d**) in the presumed downrange directions. Spherical harmonic degrees 1–2 were removed for the SPA basin (see Supplementary Information for crustal thickness maps and profiles without removing degrees 1–2) and younger basins were masked out. Source data

Basins with confirmed trajectory directions also exhibit steeper transitions in crustal thickness and topography at the uprange ends and more gradual transitions at the downrange ends, as predicted by laboratory experiments and numerical models^28,30^. This asymmetry is clearly demonstrated at the Hellas basin, with uprange and downrange gradients in crustal thickness of 0.084 and 0.034 km km^−1^, respectively (Fig. 2), and topographic gradients of 0.024 and 0.009 km km^−1^, respectively. For the SPA basin, the northern end exhibits a steeper transition than the southern end in crustal thickness (0.16 versus 0.016 km km^−1^, respectively) and topography (0.018 versus 0.003 km km^−1^, respectively), confirming the southward impact trajectory. However, both pre-impact and post-impact processes can affect the crustal thickness gradients (see Supplementary Discussion). Crustal thickness profiles of the SPA basin also reveal a local increase in crustal thickness by about 13 km immediately south of the basin beyond the downrange rim (Fig. 2d), consistent with the model-predicted ejecta distribution^3^.

## Basin ejecta composition

**Fig. 3:**
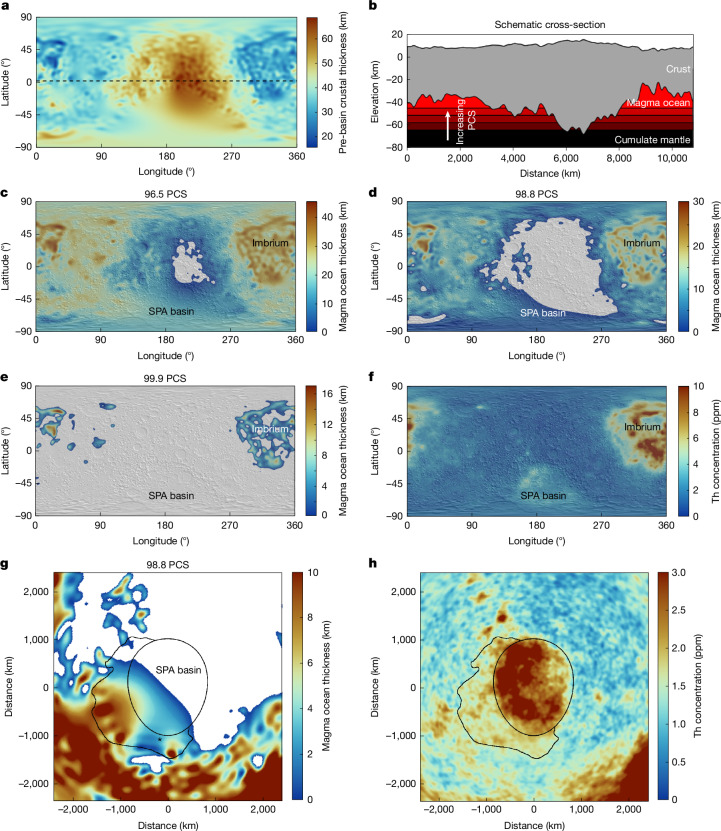
Compositional evidence for excavation of the late-stage lunar magma ocean. **a**, Model of the pre-SPA basin crustal thickness distribution (global map, centred on the far side). **b**, Schematic of the distribution of the magma ocean trapped beneath areas of thinner crust during progressive crystallization through a transect along the dashed line in **a**. **c**–**f**, Models of the thickness of the residual magma ocean (**c**–**e**) at two stages of crystallization representing 96.5 PCS (**c**), 98.8 PCS (**d**), at which point the SPA basin-forming impact is proposed to have occurred, and 99.9 PCS (**e**), matching the final distribution of KREEP-rich material (**f**). The locations of the SPA basin and Imbrium are labelled in **c**–**f**. **g**,**h**, Remnant magma ocean thickness at the SPA basin at the proposed time of impact at 98.8 PCS, for which the residual magma ocean is present only beneath the southwestern half of the basin (**g**), matching the distribution of Th-rich material in the ejecta blanket (**h**) (outer outlined zone; inner ellipse based on the rim fitting; projection as in Fig. 1a). The location of the south pole and nearby Artemis landing sites is labeled in **g** (asterisk). Source data

The SPA basin-forming impact excavated deeply below the crust^2,3,36,37^ and so the composition of the basin ejecta provides a means of examining the three-dimensional structure of the Moon at the time of impact. The high concentrations of Th and Ti in the basin interior (Fig. 3f) probably reflect a mixture of impact ejecta from the collapsed rim^3,11,37^ and differentiated impact-generated melts sourced from depths up to 200–400 km including the Ti-rich cumulates^12,38^. Although previous work interpreted the highest Th concentrations within the basin as the collapsed ejecta^11^, gravity data revealing the distributions of both the mantle-bearing ejecta and the central melt sheet show that the highest Th concentrations are contained largely within the melt sheet^39^ (see Supplementary Discussion). Here we focus on the material beyond the topographic rim as most representative of ejecta that is free from contamination from the central melt sheet. This ejecta should be focused in the downrange and cross-range directions^3,36,37^.

To the north of the basin, in which a northward trajectory would predict a kilometres-thick Th-rich ejecta blanket extending nearly 1,000 km from the rim^3^, there is no excess in either Ti or Th apart from unrelated Th-rich spots resembling those found elsewhere in the lunar highlands^40^. By contrast, a broad lobe of increased Th concentrations is seen extending west and south of the basin (Fig. 3f; see Methods), in which the western half of the ejecta blanket would be found for a southward trajectory. Here a Th concentration of 1.79 ± 0.03 ppm (mean and 1 standard error range) in the southwest ejecta of the SPA basin is much greater than both the typical far-side highland concentration of 0.95 ± 0.02 ppm and the equivalent area east of the basin at 1.17 ± 0.03 ppm, but is lower than the basin centre at 2.78 ± 0.05 ppm. Similar patterns of enrichment are found for FeO, consistent with a general trend of decreasing Th, FeO and mafic mineral abundance with distance south of the basin^41^. Although the basin centre is strongly increased in Ti (0.55 ± 0.03 wt%), there is no notable excess in titanium in either the west (0.22 ± 0.02 wt%) or the east (0.28 ± 0.02 wt%) ejecta blankets relative to the highlands (0.26 ± 0.02 wt%). Although the surface concentrations of Th and other elements have been affected by a long history of impacts throughout this region, post-SPA basin impacts cannot explain either the high Th concentration of the southwest ejecta or the low Th concentration of the northeast ejecta (see Supplementary Discussion).

## Magma ocean source for the west ejecta

**Fig. 4:**
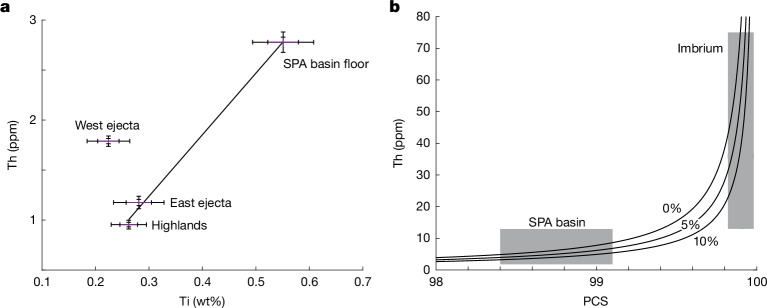
Observed concentration of Th and Ti around the SPA basin and expected evolution of Th concentration during magma ocean crystallization. **a**, Concentration of Th as a function of Ti in areas of interest within and around the basin (error bars indicate the 1 and 2 standard error ranges). The west ejecta blanket does not lie on the line encompassing the other three zones. **b**, Predicted Th concentration as a function of PCS of the magma ocean for assumed trapped melt fractions within the cumulate mantle ranging from 0% to 10%. Constraints from the ejecta of the SPA basin and Imbrium basin are indicated by grey boxes. Source data

In terms of Ti and Th abundance, the west ejecta cannot be composed of a simple mixture of the basin floor and far-side highlands materials (Fig. 4a). These observations require mixing of at least three different reservoirs to explain the surface compositions: Th-poor and Ti-poor highland crustal material, a Th-rich and Ti-rich reservoir dominating the basin floor and a Th-rich but Ti-poor reservoir sampled in the west ejecta. The east ejecta is probably dominated by excavated crustal material together with mantle cumulates, as supported by the observation of pyroxenes in one part of the eastern basin rim^3^, although that area was also affected by Apollo basin ejecta^11^. By contrast, the west ejecta must have sampled a separate Fe-rich and Th-rich reservoir that was not similarly enriched in Ti. The most likely candidate for this Th-enriched reservoir is the late-stage magma ocean^11^. The Ti content of the lunar magma ocean progressively increased until ilmenite or Ti-spinel^42^ began to crystallize at >90 percent solidification (PCS), after which the concentration decreased in the more evolved stages. By contrast, the concentration of Th continued to increase throughout magma ocean crystallization, as it was efficiently excluded from all of the crystallizing mantle and crustal mineral suites. Although FeO is removed in proportion with Ti, it is present in much higher concentrations in the magma ocean and is less affected by ilmenite crystallization. Thus, the latest stage magma ocean liquids were strongly enriched in Th relative to Ti.

The excavation of these late-stage magma ocean liquids only beneath the southwestern half of the basin is consistent with a model of the evolving spatial extent of the magma ocean during crystallization. For a crust of variable thickness floating on a magma ocean above a hydrostatic solid interior, the residual magma ocean would naturally be thicker where the crust was thinner^43^. As magma ocean crystallization continued, areas of thicker crust would have become grounded on the underlying solid cumulate mantle, resulting in a magma ocean that pinched out to zero thickness where the crust was thickest, with continued crystallization progressively concentrating the liquid where the crust was thinnest. Although the mechanism for forming the lunar crustal asymmetry remains uncertain^23^, a natural consequence of this asymmetry is that the final magma ocean liquids will reside beneath the thin crust of the near-side PKT as long as the asymmetry was established by the late stages of magma ocean crystallization^43^.

We model this process by first removing the effects of impact basins from a model of the pre-mare crust^44^ to represent the crust before the SPA basin-forming impact (Fig. 3). We then track the thickness distribution of the magma ocean during progressive crystallization. When crystallization had proceeded to 95.2 PCS, the far-side highlands became grounded on the underlying solid interior. By 99.90 PCS (allowable range 99.82–99.98 PCS), the magma ocean was largely concentrated within the PKT, with a mean regional thickness of 3.1 km and a peak thickness of 14.4 km. Between these extremes, at 98.8 PCS (range 98.4–99.3 PCS), a discontinuous magma ocean would have extended throughout the near side and parts of the far side, with a typical thickness of 5 km beneath what is today the southwestern half of the SPA basin, in which increased Th is found in both the basin floor and adjacent ejecta blanket, but residual magma would have been absent beneath the northeastern half of the basin (Fig. 3g). An impact at this stage would have excavated KREEP-rich late-stage magma ocean liquids only in the southwest, generating an asymmetric Th-rich ejecta blanket as observed. This scenario is supported by recent evidence from Chang’e-6 samples that the mantle beneath the northeastern portion of the SPA basin did not contain a substantial KREEP component^45^. The alternative scenario of asymmetric excavation of a uniformly Th-enriched upper mantle beneath a crust of variable thickness can be ruled out, as this cannot explain either the high concentrations of Th in the southwest ejecta or the low concentrations of Th in mantle-bearing ejecta east of the basin (see Supplementary Discussion).

The excavation of a KREEP-rich reservoir by first the SPA basin-forming impact and later the near-side Imbrium basin-forming impact^46^ provides two data points to constrain the evolution of the magma ocean in space, time and composition. On the basis of the surface abundances, the Th concentration in the residual magma ocean excavated by the SPA basin was 1.8–12.8 ppm, depending on the mixing ratio between excavated magma ocean and crustal material (see Methods). Later, after the establishment of the near-side PKT, the concentration of Th in the Imbrium ejecta reveals the concentration in the final solidified KREEP reservoir of 13–75 ppm, depending on the mixing of materials in the ejecta^47^.

These estimates can be compared with the predicted concentration of Th in the residual magma ocean liquids during crystallization (see Methods). Th is strongly partitioned into the melt during crystallization^48^, such that the dominant control over the concentration of Th in the residual liquids is the fraction of the melt that becomes trapped in the interstices between the cumulates. At 98.8 PCS, the Th concentration in the evolving liquid ranges from 2.8 to 4.3 ppm for entrapped melt fractions ranging from 0 to 10%, in agreement with the constraints from the SPA basin ejecta (Fig. 4b). At 99.9 PCS, the Th concentration ranges from 20 to 40 ppm, in agreement with the constraint for the PKT from the Imbrium ejecta.

The presence of a magma ocean layer at depth at the time of impact would have affected the morphology of the resulting basin. The topographic transition between the basin centre and the surroundings for the SPA basin is more gradational than observed in smaller lunar basins, consistent with the effects of a thin magma ocean on basin formation^6^ and relaxation^49^. Moreover, the Bouguer gravity transition along the western rim is more gradational than in the east (Fig. 1a), consistent with the effects of a local magma ocean. Partial excavation of late-stage magma ocean liquids by the impact is also consistent with chlorine isotopic evidence for late-stage degassing of the magma ocean associated with at least one large-scale crust-breaching impact^50^.

## Implications for early lunar evolution

This work has important implications for our understanding of the SPA basin, the early evolution of the lunar crust and magma ocean and the bombardment history of the Solar System. Our results indicate that the SPA basin formed from an impact on a southward trajectory at a time when the Moon still had a partial, discontinuous magma ocean. The compositions of the Ti-rich and Th-rich basin interior and the Th-rich ejecta indicate that, even at this late stage of magma ocean crystallization, there was vertical segregation of the residual KREEP-rich magma ocean liquids from Ti-rich cumulates rather than a single Ti-rich and KREEP-rich reservoir. The distribution and concentration of Th-rich ejecta at the SPA basin and Imbrium together support a model of passive concentration of the final liquids of the magma ocean beneath the thin crust of the near side^43^, with a separate process required to bring the Ti-rich cumulates to the near side^51,52^.

Models^5^ predict magma ocean crystallization to take about 50–200 Myr, with the final 1% taking about 10–75 Myr owing to the decreasing crystallization temperature with evolving composition. As a result, the latest stages of magma ocean crystallization are the most likely time for the SPA basin-forming impact. This timing supports the possibility of a SPA basin-forming impact several tens of millions of years before the completion of magma ocean crystallization and the establishment of the final KREEP reservoir at 4.34–4.37 Ga (ref. ^10^). This age constraint is consistent with sample-based ages and crater retention model ages of the basin^14–17^ of 4.31–4.39 Ga and with dynamical simulations suggesting that a basin age of 4.36–4.41 Ga would support a declining bombardment scenario in which there was an exponential decrease in the rate of impacts after the formation of the Solar System^4,53^. However, the declining bombardment scenario requires complete relaxation of a population of earlier basins^53^, whereas the preservation state of this basin formed over a partial magma ocean suggests that complete relaxation of a comparable basin would require a substantially hotter or thicker crust^49,54^. Alternatively, a 4.43 Ga age for KREEP solidification^55^ and older age for the basin may support the need for a later increase in impact flux, referred to as a late heavy bombardment.

This new understanding of the basin has important implications for the upcoming robotic and human exploration of the lunar south pole, a key goal of which is to sample the ejecta of the SPA basin^41^. Proposed south polar Artemis landing sites are now seen to be situated within the Th-rich ejecta blanket at the downrange end of the basin. Thus, the rocks sampled by Artemis may constrain not only the age of the basin and history of lunar bombardment but also the composition of the late-stage magma ocean and timing of its solidification.

## Methods

### Determination of basin outlines

In this study, we identify the basin outline using the discrete transition in Bouguer gravity^56,57^ (the gravity anomaly corrected for the effects of topography) and topography at the outer edge of the central zone in which the crust has been thinned by the impact. The outlines of the comparison basins were identified using the radial distance at which the contrast from the basin centre to exterior (in Bouguer gravity or topography) reached 50% of the maximum value. This choice makes the radius relatively insensitive to data resolution and also less sensitive to the natural variability in the data than identifying points closer to the geological rim of the basin at a greater radial distance at which the quantity of interest is closer to the surrounding value. As a result, basin dimensions are smaller than previously published values in which the rim is typically defined using the high point in topography outside the steep transition leading into the basin centre. For multiring basins, our basin dimensions are much smaller than the outer ring diameters but are comparable with the diameters of the positive Bouguer anomalies^58^. For basins that are largely isostatically compensated, topography, Bouguer gravity and crustal thickness all yield similar results. However, topography is more strongly affected by external processes such as impacts and erosion, and crustal thickness models typically require strong filtering to stabilize the solution, thereby decreasing their resolution. For these reasons, Bouguer gravity is taken as the preferred dataset. For the buried Utopia impact basin, the proximity of the basin to the dichotomy boundary complicates the selection of the outline and we manually select the Bouguer gravity value to use as the threshold at a value closer to the basin floor to mitigate this effect. For all basins, we mask out areas that are affected by other later processes (for example, impact craters superposed on the Crisium and Smythii rims, volcanic features and erosion on the rim of Hellas and the downrange tail region in the south and tectonic modification on the northern edge of Sputnik).

For the SPA basin, the above criterion yields a very irregular outline. The topography of the basin has been heavily modified by subsequent cratering, making the topographically defined basin shape unreliable. The Bouguer gravity and crustal thickness signature of the basin provides a better outline, but the transition between the basin floor and surrounding highland crust is slightly more gradational and irregular, particularly in the west, leading to large outward excursions in the rim location defined using a simple threshold. As discussed in the text, this may reflect the effect of the subsurface magma ocean on the collapse of the transient cavity in the western half of the basin, as predicted by models^6^. The western outline in Bouguer gravity is similarly affected by the large linear gravity anomalies related to intrusive bodies^31,59^. Moreover, the superposition of the basin on the global crustal and Bouguer gravity asymmetry has the potential to introduce errors in a simple threshold-based approach.

Instead, for tracing the outline of the SPA basin, we use gravity gradients, which we calculate as the maximum amplitude eigenvalue of the tensor of second horizontal derivatives of the Bouguer potential^31,60^. The short-wavelength gravity gradients are strongly sensitive to long-wavelength variations in surface topography owing to the strong attenuation/amplification of the gravity anomalies with upward/downward continuation, resulting in much stronger values in areas of high topography when evaluated at a constant radius. This effect is removed by calculating the gravity and gravity gradients at the local surface of a smooth (spherical harmonic degree 30) topography model, so that the gravity gradients are everywhere evaluated at the local surface^61^. In the gravity gradients, sets of discontinuous arcuate features circumferential to the basin define the basin outline. We identify the gravity gradient ring most closely matching the point at which the Bouguer anomaly reaches half of its maximum contrast in the eastern portions of the basin and trace this feature around the basin. In the northeastern quadrant of the basin, the gravity gradient ring most closely matching the Bouguer threshold approach takes a step inward towards the basin centre, leading to a more irregular outline, whereas in the northwest, the Bouguer threshold deviates out away from the basin rim. Our preferred outline tracing is based on a compromise between proximity of the gravity gradient feature to the 50% Bouguer threshold, continuity of the gravity gradient arcs and a smoothly varying basin rim.

**Extended Data Fig. 1:**
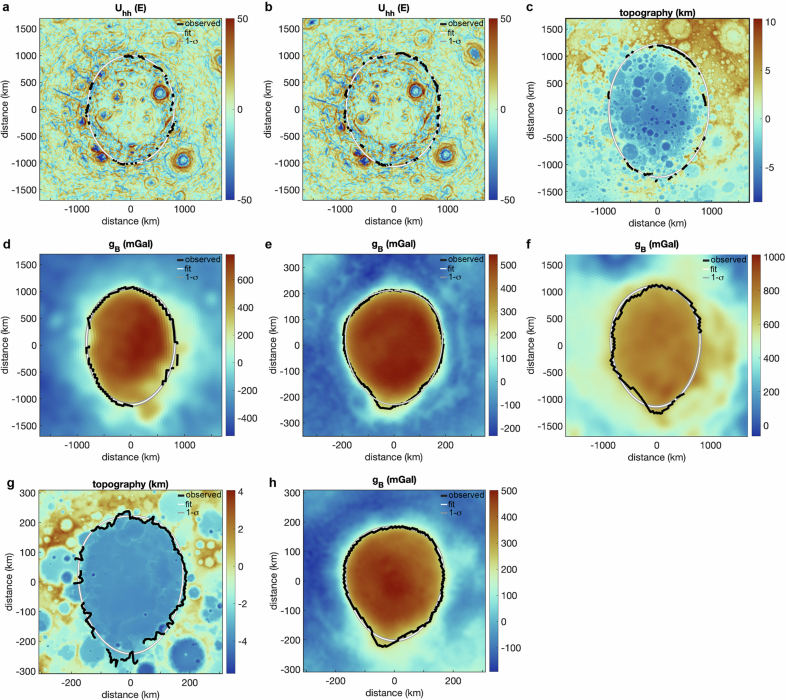
Further basin outline choices and best-fit tapered ellipses. **a**–**h**, Panels show two further outline tracings based on Bouguer gravity and gravity gradients for the SPA basin (**a**,**b**, corresponding to fits 2 and 3 in Extended Data Table 1), an extra outline tracing for the SPA basin based on topography (**c**, fit *z*, 2 in Extended Data Table 1), analyses of the Hellas (**d**), Crisium (**e**), Utopia (**f**) and Smythii (**h**) basins in Bouguer gravity and an analysis of the Smythii basin in topography (**g**). All panels are in basin-centred polar projections with the inferred downrange direction to the bottom.

As well as the gravity-based outline above and the topographic outline based on discontinuous massifs and scarps discussed in the main text, we also evaluate two different choices of basin outline for the SPA basin using gravity gradients and Bouguer gravity (Extended Data Fig. 1). The second outline is designed to more closely follow the 50% Bouguer gravity threshold, resulting in a more irregular basin rim with a distinct shift in radius in the eastern basin rim. The third outline selects the outermost prominent gravity gradient ring, although this seems to highlight an outer ring in the eastern basin compared with the western basin. We also test one extra topographic outline tracing for the SPA basin that stays further from the basin centre in the southern part of the basin. Ultimately, the precise choice of outline for the SPA basin is uncertain, but all choices taper southward, as shown in the next section.

### Basin outline fitting: tapered ellipse

With a discontinuous and irregular basin outline, it is not possible to simply measure the tapering of the basin outside some model of a fit to the shape of the outline. To fit the basin outlines, we first identified the best-fit centre by fitting the radial distance of the basin outline as a function of azimuthal angle as defined from the approximate basin centre with a Fourier transform and using the first Fourier mode to determine the offset between the true basin centre and the initial choice. The second Fourier mode reveals the long-axis orientation. We then use a Metropolis–Hastings Markov chain Monte Carlo algorithm to iteratively adjust the outline fit based on the misfit to the data.

To fit the data to a simple tapered ellipse, we use a shape defined by:

$$y=a\cos (\theta )$$

$$x={\left(\left(1-\frac{{y}^{2}}{{a}^{2}}\right)\frac{{b}^{2}}{1+ky}\right)}^{1/2}$$

with the basin centred at the origin, in which |*k*| < 1/*a*. For the case of *k* = 0, this equation defines a simple ellipse with semimajor axis *a* and semiminor axis *b*. Positive and negative values of *k* define tapers in opposite directions and thus provide a means of evaluating the probability of a northward taper and northward projectile direction for the SPA basin. In the Markov chain Monte Carlo algorithm, *a*, *b* and *k* are varied, as well as the basin centre point location. This approach allows for a simple elliptical basin with no preference in tapering or impact direction. The basin tapering factor *f* is calculated by taking the difference between the basin width at the points midway between the basin centre and the uprange/downrange ends and dividing by the along-track distance between these points. Thus, this tapering represents the gradient in basin width of the middle 50th percentile of the basin. Fits were obtained for all basins discussed in this work (Extended Data Fig. 1). The mean and 1*σ* ranges in parameters were taken from the posterior distributions of the model (Extended Data Table 1) after 25,000 iterations with a burn-in period of 2,000 iterations. As well as the tapered ellipse, we also fit the basin outlines using a geometric model based on the physics of the formation of a tapered elongated impact basin from an oblique impact (see Supplementary Discussion).

### Surface composition

**Extended Data Fig. 2:**
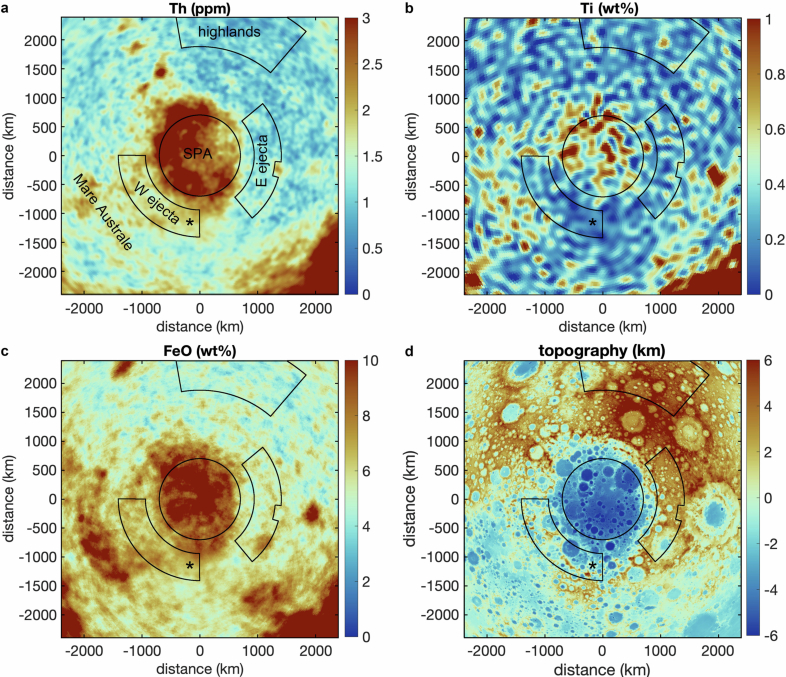
Surface composition within and around the SPA basin. **a**–**d**, Distribution of Th (**a**), Ti (**b**) and FeO (**c**) around the SPA basin, showing the domains over which the surface composition was averaged. The topography is shown in panel **d** for reference. The south pole and surrounding Artemis landing site region is indicated by an asterisk.

We analyse the surface composition using elemental data derived from the low-altitude, high-resolution (0.5°) Lunar Prospector Gamma Ray Spectrometer (GRS) data^40,62,63^. All data are reprojected in a basin-centred polar projection. Regions of interest are defined on the basin floor, in the western ejecta blanket, in the eastern ejecta blanket and in the highlands north of the basin using sectors in an axisymmetric reference frame (Extended Data Fig. 2). In each region of interest, we calculate the area-weighted means and standard deviations of Th, Ti and FeO. The standard deviations are converted to standard errors (the uncertainties on the means) by scaling by *n*^−0.5^, in which *n* is the effective number of independent observations in a given area assuming a dataset resolution of 5° of arc (we use a lower resolution than the actual grid resolution to be conservative, as the data are derived from a range of measurement heights). For Ti, the data are highly pixellated, so we smooth the data using a spherical harmonic filter with a low-pass cosine transition between degrees 50 and 70 (half-wavelength resolution of about 90 km) to highlight regional trends.

Further to the west (lower left in the figures), outside the probable extent of the SPA basin continuous ejecta blanket, the Th concentration remains high within the region surrounding Mare Australe. The highest Th concentrations are found within the mare units themselves and associated cryptomare deposits^64^ and thus are unrelated to the SPA basin ejecta deposit. These areas are excluded from the analysis. By contrast, increased Th concentrations within the broad SPA basin ejecta blanket are not associated with known mare or cryptomare deposits and remain at similar higher levels through the cratered highlands in that area. To the north-northwest of the SPA basin, two small (roughly 200 km) areas of strongly enriched Th concentrations are found, one of which is associated with a known cryptomare deposit^64^. These localized features are not consistent with the expected broad distribution of a well-mixed ejecta blanket of relatively uniform composition and are more likely related to Th-rich features found globally^40^, including the Compton–Belkovich anomaly^65,66^, and to the near-side Th-rich red spots^67^, some of which are associated with local magmatic and volcanic processes. Excluding these spots, Th concentrations north of the basin are indistinguishable from the far-side highlands, as discussed in the text. A similar Th-rich spot east of the basin is also excluded from the analysis.

### Magma ocean spatial distribution

Although there are many unknowns about early lunar evolution and the development of the present-day asymmetry^23^, a simple model can account for the compositional asymmetry based on known or well-accepted aspects of lunar structure and evolution as described in the text. The Moon has an asymmetric crust that is much thicker on the far side than on the near side^21,68^ and that crust is largely isostatically supported apart from an important but small-amplitude fossil figure^69^. The crust of the Moon crystallized from a magma ocean, which caused the progressive enrichment of incompatible elements including KREEP in that magma ocean. From these very simple aspects of lunar structure and evolution, the concentration of the final dregs of the magma ocean on the near side can be seen to be a natural consequence of the crustal asymmetry.

For a crust and mantle in vertical hydrostatic equilibrium, the remnant magma ocean trapped between the crust and cumulate mantle at any point will be thicker where the crust is thinner^43^. This simple process can account for the near-side KREEP terrane without invoking any further geodynamic complexities, although it does not explain the formation of the crustal asymmetry to begin with nor the later concentration of Ti-rich cumulates on the near side, which required an extra process^51,52,70,71^. The fundamental assumption made by this model is that the crustal asymmetry was largely established by the late stages of magma ocean crystallization, but it does not otherwise depend on the mechanism for generating the asymmetry.

We model the lateral distribution and thickness of the late-stage magma ocean assuming that the crust and mantle are in vertical hydrostatic equilibrium. We assume that the long-wavelength crustal thickness patterns outside the main impact basins were established during crustal formation. The modelled crustal thickness within the PKT is affected by the presence of the maria, so we begin with a model of the lunar crust before the maria^44^, which has a thinner crust within the PKT than models that do not account for the presence of the maria^68^. We interpolate the crustal thickness across the 38 largest impact basins^58^, including the SPA basin itself, using an inverse-distance-squared algorithm. For all basins smaller than Serenitatis, there is commonly a pronounced thickening of the crust by ejecta outside the central positive Bouguer anomaly and we scale the positive Bouguer anomaly diameter by a factor of two before basin removal. For the SPA basin, we scale the diameter by 1.2 to avoid any rim and ejecta effects. After the interpolation, the base of the crust is filtered in the spherical harmonic domain with a low-pass cosine taper between degrees 45 and 60 to remove short-wavelength structures that are probably of later origin and to smooth out the interpolation boundaries. We assume that the base of the magma ocean is a spherical interface until it intersects the base of the crust, ignoring the small effects of rotational-tidal distortion that would affect the top of the mantle and base of the crust similarly.

**Extended Data Fig. 3:**
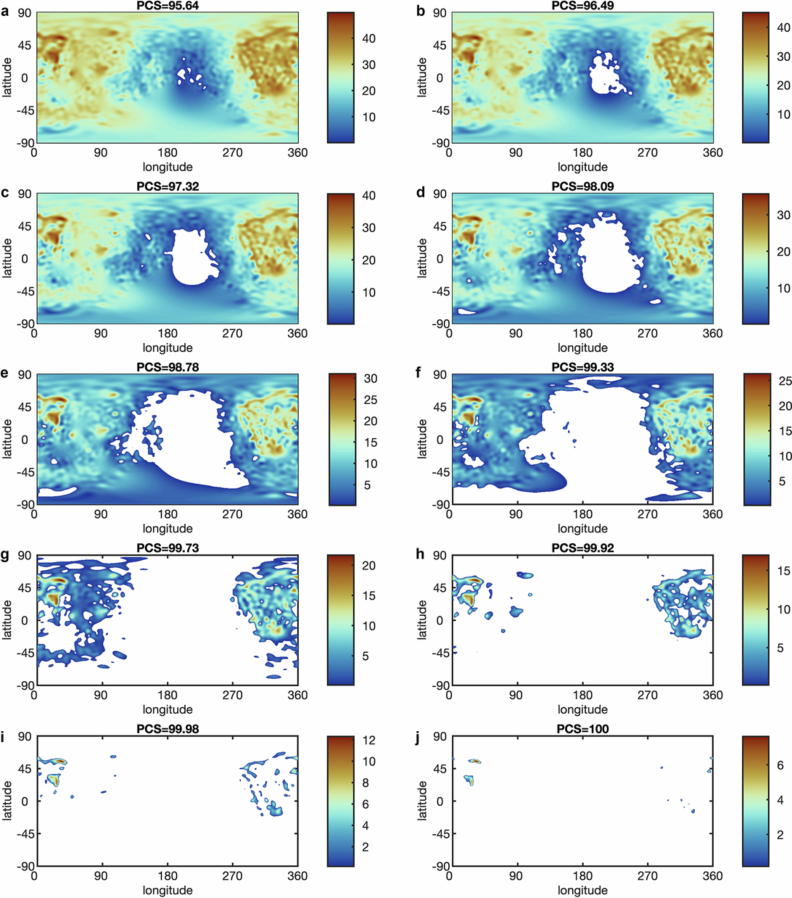
Residual magma ocean thickness as a function of PCS for late-stage progressive crystallization, assuming continued crustal floatation. **a**–**j**, Areas in white have no magma ocean because the crust has grounded on the solid cumulate mantle. Successive panels increment in constant steps in maximum magma ocean thickness, resulting in a variable step size in PCS.

We then map out the magma ocean thickness for a range of basal interfaces in 0.1-km increments beginning at the point at which the thickest crust first comes into contact with the underlying cumulates and compute the corresponding magma ocean volume and PCS (Extended Data Fig. 3). At these late stages of crystallization, the bulk cumulate mixture (including mafic and feldspathic minerals) is negatively buoyant^42^. It is unknown whether these minerals segregate by density and the floatation crust continues to thicken or whether the bulk crystal mixture sinks. We consider both scenarios, assuming that plagioclase makes up 20% of the column of crystallizing minerals in each increment for the former case^42^. For models in which the crust continues to thicken during these late stages, a model beginning with the present-day crustal thickness will end with a crust approximately 7 km too thick where the crust is thinnest. We iterate the model, adjusting the starting thickness to end up with the desired final pre-SPA basin crustal thickness model. The nominal model assumes a magma ocean depth of 1,000 km (bottom radius at 737 km) but we also consider magma oceans extending to the core–mantle boundary at 400 km (ref. ^72^). The assumed initial thickness of the magma ocean only affects the calculation of the PCS at each step in the model. The nominal model assumes that the crustal thickness generated at each incremental step of crystallization is 20% of the crystallized magma ocean, but we also consider the case in which no further crust is generated because the mixed cumulates together are negatively buoyant.

We compare the predicted distribution of the magma ocean remnants to the Th anomalies in the PKT and the SPA basin, defined using the 3-ppm Th concentration contour. For the SPA basin, the distribution of Th on the basin floor as a function of azimuth is similar to that in the ejecta blanket, with the highest Th concentrations in the western half of the basin. For the SPA basin, the early stages of magma ocean crystallization will always have magma ocean beneath the Th anomalies on the basin floor, but the impact occurred at a transitional time in which the magma ocean was pinching out to zero thickness in the northeast of the basin. Many areas outside the basin probably did have remnant magma ocean at the time of impact and so cannot be used to evaluate the models. We compute the areal fraction of the high-Th zone within the basin underlain by magma ocean and identify the model with 50% coverage as the optimal model and the middle 50th percentile as the acceptable range (Extended Data Table 2). A qualitative comparison of the distribution of magma ocean relative to the distribution of Th-rich ejecta outside the basin yields similar results. For the PKT at the latest stage of magma ocean crystallization, the desired solution will have magma ocean below the PKT but not beneath the surroundings and thus models are penalized for both the lack of magma ocean within the PKT and the presence of magma ocean outside it. The optimal model has the best areal fit to the observed PKT and we identify the range of models that differ from the best fit by less than half of the total area of the PKT as the acceptable range. In the discussion in the text, we focus on the optimal values from the nominal model with a 1,000-km-deep magma ocean and continued crustal formation, and the range from all models, yielding a PCS for the SPA basin of 98.8% (98.4–99.3%) and for the PKT of 99.90% (99.82–99.98%).

**Extended Data Fig. 4:**
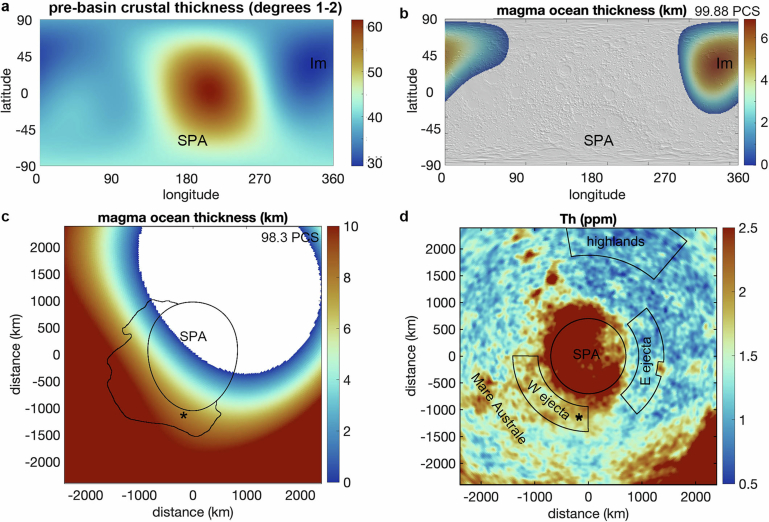
Predicted distribution of late-stage magma ocean for a simple pre-SPA basin crustal thickness model. The model shown is as in Fig. 3 but using only spherical harmonic degrees 1 and 2 in the pre-SPA basin crustal thickness model. **a**, Model of the pre-SPA basin degrees 1 and 2 crustal thickness distribution (global map, centred on the far side). **b**, Model of the thickness of the residual magma ocean at 99.88 PCS matching the final distribution of KREEP-rich material. **c**, Remnant magma ocean thickness at the SPA basin at the proposed time of impact at 98.3 PCS for this crustal thickness model, in which residual magma ocean is present only beneath the southwestern half of the basin, consistent with the distribution of Th-rich material in the ejecta blanket (**d**). In **c**, the outer outlined zone represents the ejecta, and the inner ellipse is based on the rim fitting (projection as in Fig. 1a).

The results of this study are not strongly sensitive to the details of the pre-SPA basin crustal thickness model. The topography and crustal thickness of the Moon outside the main impact basins is dominated by global spherical harmonic degrees 1 and 2 patterns^69^. The location of the SPA basin southwest of the peak far-side crustal thickness and the orientation of its long axis towards the northwest^18^ dictates that the crust will be thicker to the northeast and thinner to the southwest, as in our reconstructed pre-SPA basin crust. The lack of any strong crustal thickness anomalies on the scale of the SPA basin or smaller, apart from identified impact basins, indicates that the presence of any strong departures from the global trends in the pre-SPA basin crust is unlikely. The preservation state of the SPA basin indicates that the crustal thickness distribution has not changed appreciably since that time, apart from later formed impact basins. If we instead take only the spherical harmonic degrees 1 and 2 components of the pre-SPA basin crustal model, the late-stage magma ocean liquids still concentrate first in the southwest half of the SPA basin and later in the PKT (Extended Data Fig. 4). Other studies have attempted to reproduce the pre-SPA basin crustal thickness distribution under different sets of assumptions^73^ but still produce the northeast–southwest gradient in crustal thickness across the basin that is responsible for the predicted excavation of late-stage magma ocean liquids in our work. Any model of the pre-SPA basin crust with a gradual decrease in crustal thickness away from the peak value in the far-side highlands will yield similar results.

### Estimates of magma ocean composition based on surface Th abundance

The estimated concentration of Th in the reservoir depends on the assumptions made about the mixing of materials in the ejecta. As a lower-bound estimate on the Th concentration in the excavated reservoir at the SPA basin, we take the observed Th concentration in the west ejecta of 1.8 ppm assuming that the deepest materials are exposed directly at the surface in the overturned ejecta layer. More likely, the west ejecta blanket is a mixture of excavated crust and magma ocean liquids. Assuming that the late-stage magma ocean liquids had an FeO abundance of 25 wt% (ref. ^42^), the observed FeO excess of the west ejecta relative to the far-side highlands of 2.2 wt% is compatible with a mixture of roughly 9% magma ocean liquids with crustal material, equivalent to excavating a 4-km layer beneath the 40-km-thick crust. For that mixing ratio, the Th excess of the west ejecta relative to the far-side highlands of 0.8 ppm would correspond to a Th concentration in the underlying reservoir of 9 ppm, which we consider to be a more realistic estimate. This value is comparable with the peak Th concentration of 5 ppm on the basin floor, although the basin floor is probably dominated by the differentiated melt sheet^12,38^. Alternatively, if the ejecta includes material excavated from depths up to 80 km (ref. ^3^), including a residual magma ocean thickness of 5 km only in the southwestern half of the basin, the Th excess relative to the east ejecta (which also excavated mantle material) of 0.6 ppm corresponds to a reservoir concentration of 9.6 ppm, whereas the Th excess relative to the far-side highlands would correspond to a reservoir concentration of 12.8 ppm. Together, these considerations support a range of 1.8–12.8 ppm, with a preferred value of 9 ppm.

Later, after the establishment of the near-side PKT, the concentration of Th in the Imbrium ejecta reveals the concentration in the final solidified KREEP reservoir. Assuming that the peak Th concentrations at the surface correspond to pure KREEP places a lower bound of 13 ppm, whereas accounting for the mixing of the excavated KREEP-free crust and KREEP-rich material provides a more realistic estimate of 36 ppm and further assuming that the ejecta also mixes with target rock by ballistic sedimentation raises this estimate to 75 ppm (ref. ^47^). Similarly, scaling the Th excess of the peak concentrations in the Imbrium ejecta relative to the far-side highlands by the ratio of the FeO concentration in the late-stage magma ocean to the FeO excess in the ejecta yields a Th concentration of 28 ppm. These considerations support a range in Th concentrations of 13–75 ppm, with a preferred value of 28–36 ppm.

### Magma ocean composition and constraints on timing

The evolving concentration of Th in the magma ocean is dependent on the partitioning of Th between the melt and solid phases on crystallization. However, estimates of the partition coefficient in the range 10^−2^ to 10^−5^ (refs. ^48,74^) are effectively indistinguishable from perfect exclusion of Th from the solid phase. Instead, the partitioning of Th between the growing cumulate mantle and diminishing magma ocean is dominated by the presence of small quantities of melt trapped in the interstices of the cumulate mantle. Extraction of melt from the cumulate mantle would have been limited by percolation^75^, which becomes inefficient at melt fractions less than about 2%. Previous studies^5,42^ have typically assumed a trapped melt fraction of approximately 5%, with values as high as 20% deemed unlikely^42^. Because trapped melt leads to incorporation of incompatible elements in the cumulate mantle, these melt fractions correspond to an effective Th partition coefficient in the range 10^−1^ to 10^−2^, exceeding the partition coefficient for simple crystallization. We model the evolving Th concentration in the magma ocean as a function of PCS for melt fractions of 0, 5 and 10% and assuming an average Th partition coefficient of 5 × 10^−3^ (ref. ^48^). The initial Th concentration is assumed to be the bulk silicate Earth value of 79.5 ppb (ref. ^76^). The true bulk lunar Th concentration may differ from bulk silicate Earth depending on the relative contributions of the Earth’s crust, Earth’s mantle and projectile material to the debris disk that formed the Moon.

In discussing the timing of the SPA basin-forming impact, we use the constraint that the formation of the final KREEP reservoir in the PKT was largely complete by 4.34–4.37 Ga, as supported by several studies (see review in ref. ^10^). However, geochronological estimates based on unshocked zircons indicate the survival of some residual magma ocean beneath the near side until about 3.90 Ga (refs. ^77,78^). An extended duration for some fraction of the magma ocean on the near side is expected owing to the high concentrations of heat producing elements ultimately concentrated in the PKT^77^, but this does not affect the interpretations or conclusions of this work.

## Online content

Any methods, additional references, Nature Portfolio reporting summaries, source data, extended data, supplementary information, acknowledgements, peer review information; details of author contributions and competing interests; and statements of data and code availability are available at 10.1038/s41586-025-09582-y.

## Supplementary information

Supplementary Information Supplementary Discussion, Figures, Tables and References

## Source data

Source Data Fig. 1

Source Data Fig. 2

Source Data Fig. 3

Source Data Fig. 4

## Figures and Tables

**Extended Data Table 1:**
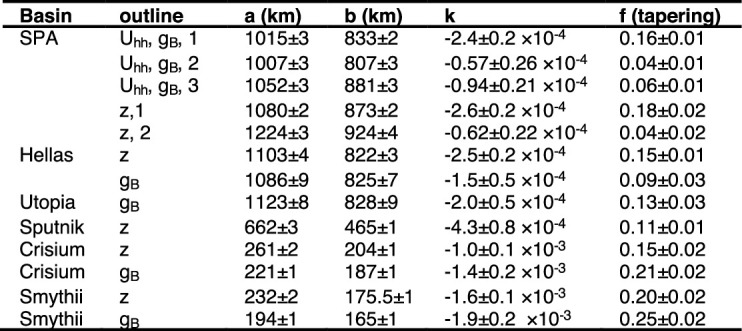
Monte Carlo model results

Mean values and 1*σ* range of the posterior distribution of key parameters from the Monte Carlo model fitting a tapered ellipse to the impact basin outlines. Several outline choices and fits are included for the SPA basin (option 1 represents the preferred choice discussed in the main text for both gravity and topography). Values include the semimajor and semiminor axes (*a*, *b*), the *k* value from the tapered ellipse equation and the tapering factor *f* calculated as the gradient in width over the middle 50% of the basin.

**Extended Data Table 2:**
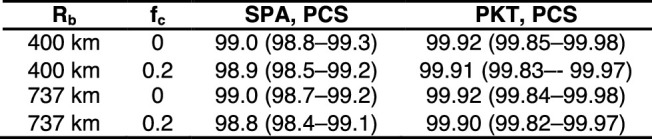
The PCS of the magma ocean matching the distribution of Th on the floor of the SPA basin and within the PKT

Optimal value and acceptable range are given as a function of the radius of the base of the magma ocean (*R*_b_) and the fraction of crystallized minerals that float to contribute to continued crustal thickening (*f*_c_). The optimal value is the best-for for the nominal model including continued crustal floatation, whereas the acceptable range includes a wider range of fits and models with and without continued crustal floatation.

## Data Availability

Gravity, topography and elemental abundance data are available on the NASA Planetary Data System Geosciences Node website: https://pds-geosciences.wustl.edu/dataserv/moon.html. Source data are provided with this paper.

## Code availability

The code used to generate models of magma ocean thickness and composition is available at Zenodo (10.5281/zenodo.16816551)^79^.
