# Supplementary material for: Southward impact excavated magma ocean at the lunar South Pole–Aitken basin
Source: Nature. 2025 Oct 8;646(8084):297–302. doi: 10.1038/s41586-025-09582-y (PMC12507655; doi:10.1038/s41586-025-09582-y)
Supplement: Supplementary file 1 — Supplementary Discussion, Figures, Tables and References [file 41586_2025_9582_MOESM1_ESM.pdf]

nature portfolio

https://doi.org/10.1038/s41586-025-09582-y

Supplementary information

Southward impact excavated magma ocean at the lunar South Pole–Aitken basin

In the format provided by the
authors and unedited

Supplementary Information for:

Southward impact excavated magma ocean at the lunar South Pole-Aitken basin

Jeffrey C. Andrews-Hanna[a,b*], William F. Bottke[c,b], Adrien Broquet[d], Alexander J. Evans[e], Gabriel Gowman[a,b], Brandon C. Johnson[f,g,b], James T. Keane[h,b], Janette N. Levin[e], Ananya Mallik[i], Simone Marchi[c,b], Samantha A. Moruzzi[a], Arkadeep Roy[i], Shigeru Wakita[f,b]

[a]*Lunar and Planetary Laboratory, University of Arizona, 1629 E. University Blvd., Tucson, AZ 85721, United States*
[b]*Center for Lunar Origin and Evolution, NASA Solar System Exploration Research Virtual Institute (SSERVI)*
[c]*Southwest Research Institute, Walnut St, Suite 300, Boulder, CO 80302, United States*
[d]*Institute for Space Research, German Aerospace Center, DLR, Berlin, Germany*
[e]*Department of Earth, Environmental, and Planetary Sciences, Brown University, Providence, RI, 02912, United States*
[f]*Department of Earth, Atmospheric, and Planetary Sciences, Purdue University, West Lafayette, IN, 47906, United States*
[g]*Department of Physics and Astronomy, Purdue University, West Lafayette, IN, 47906, United States*
[h]*Jet Propulsion Laboratory, California Institute of Technology, 4800 Oak Grove Drive, Pasadena, CA 91107, United States*
[i]*Department of Geosciences, University of Arizona,* 1040 E 4th St., Tucson, AZ 85715, *Tucson, AZ 85719, United States*
[*]*to whom correspondence should be addressed: jcahanna@arizona.edu*

Supplementary Discussion
Supplementary Figures 1-5
Supplementary Table 1
Supplementary References

Supplementary Discussion

Tapered craters and basins

It has long been recognized that craters formed in highly oblique impacts are elongated in the direction of projectile motion, and those formed in the most oblique impacts exhibit a more complicated morphology including downrange tapering, asymmetric profiles in cross-section, and downrange scour marks or "sibling" craters[28,80]. At the scale of simple and complex craters, these morphologies are only found for impact angles <5°, making up <1% of the crater population[28,81,82]. Although elongated simple and complex craters on planetary surfaces are common[81-83], a consistent tapering is not universally observed, perhaps in part because smaller impact craters are not generally affected by the curvature of the planetary surface. A noteworthy exception is the Orcas crater (patera) on Mars, which is a highly elongated crater (370 × 140 km) that tapers prominently toward the south. A crater of this scale is only expected to be significantly elongated for impact angles <5° (ref. [81]), but the crater itself spans 6° of arc in the long dimension and thus would be strongly affected by the curvature of the surface despite its small size.

At much larger sizes, elongated and asymmetric structures become increasingly common. The majority of giant impact basins are elongated[20] and our analysis here shows that all currently known elliptical impact basins exhibit a tapered shape toward one end of the long axis (Hellas, Utopia, SPA, Crisium, Smythii, and Sputnik). Even the proposed Borealis basin on Mars tapers slightly in one direction, interpreted as a possible outcome of the development of a partial ring structure[84]. The most studied[1,34] of the elliptical basins is Crisium on the Moon (Fig. 1e). Crisium has long been recognized as tapering toward the east[1,34]. An eastward moving projectile has been supported based on the elongated and tapered shape, the gap or breach in the peak ring toward the east as could occur from the downrange impact of a decapitated projectile, variations in the relief along its rim, and the distribution of its ejecta[1,34]. The Hellas impact basin on Mars has also been noted as an elongated impact basin in previous work. An eastward trajectory has been supported based on the distribution of a population of high-relief massifs extending ~700 km from the eastern end of the basin[32,33], which is also in the direction of basin tapering. These massifs have horizontal scales of 10's of kilometers and typical relief of several kilometers. Remote sensing data indicates that these massifs have a unique composition consistent with the excavation of a feldspathic lower crust by the impact[85].

Insight into the cause of the downrange tapering comes from laboratory experiments and numerical models. At laboratory scales, tapered and complicated crater shapes form from highly oblique impacts when part or all of the projectile ricochets off the surface and re-impacts further downrange[28]. Although the downrange tapering is similar to what is found in nature at larger scales, the ricochet process is not directly applicable to planetary-scale impacts. The process is somewhat analogous as the smaller fragments impacting downrange have less mass/energy and produce smaller craters, leading to a downrange tapering in the overall structure. In small-scale experiments with curved target surfaces and larger scale numerical models, decapitation of the projectile can occur, in which a portion of the projectile shears off and continues on its trajectory to impact further downrange, creating distinctive crater and basin shapes that taper down range and exhibit extended tails[30].

At the basin scale, projectile decapitation may also play a role[30]. However, at these scales the curvature of the planetary surface also becomes important[30] as the basin diameter becomes an appreciable fraction of the planetary radius[20], potentially explaining the preponderance of tapered outlines in giant elongated impact basins. Three-dimensional numerical models of the formation

of the South Pole-Aitken basin predict downrange tapering[24,36]. Early CTH models of the impact of a 200-km-diameter projectile at a 45° angle predicted a tapered impact basin, though the resulting basin shape was elongated perpendicular to projectile motion and pinches inward at the up-range end in contrast to observed basin shapes[24]. A subsequent model was presented in a manner that does not permit evaluation of the basin shape[3]. More recent CTH models of the impact of a 360 km-diameter projectile impacting at 30° angle resulted in a basin somewhat smaller and less elongated than observed (aspect ratio of 1.08 vs. 1.2–1.3), but clearly elongated and tapering in the downrange direction[36]. Ongoing iSALE3D modeling of a 260-km-diameter differentiated body impacting at a 30º angle predict a basin that tapers in the downrange direction, related to the downrange impact of the core of the differentiated projectile[37]. A challenge for future modeling studies is the observation that all of the largest impact basins are both tapered and strongly elongated (aspect ratios 1.2-1.43; ref. [20]), while models generally predict less elongated impact basins even at low angles (aspect ratios <1.1).

Thus, while continued work is needed on the formation of elongated and tapered craters and basins, evidence from laboratory experiments, numerical models, and planetary observations all support a tapering of the crater or basin shape in the downrange direction at scales ranging from centimeters to thousands of kilometers. Each of the processes discussed above may play a role in the generation of these tapered impact structures, and the relative importance of each may vary with scale and setting.

Other factors may also affect the shape of impact basins. Variations in crustal thickness may affect the basin excavation process. However, the crustal thickness distribution around Hellas is effectively uniform[86], so pre-existing gradients in crustal thickness cannot be the cause of that tapering. Similarly, neither Crisium nor Smythii on the Moon taper in the direction of a regional crustal thickness gradient[68], and there is no evidence for a gradient in topography or shell thickness around Sputnik basin on Pluto[87]. The direction of elongation and tapering of SPA is oblique to the pre-existing crustal thickness gradient. The heat flow at the time of impact also affects basin formation[2,6,88]. For SPA, the narrower end of the basin is both closer to the high heat flow Procellarum KREEP terrane and over thinner crust where remnant magma ocean is more likely, but high heat flow and the presence of a magma ocean should result in a widening of the basin[2,6,88] opposite what is observed. While Crisium does narrow in the direction away from the PKT (toward cooling temperatures), geological evidence supports tapering in the downrange direction[1]. There is no data to support substantial gradients in heat flow for other tapered basins (e.g., Hellas on Mars or Sputnik on Pluto). Instead, the preponderance of observational, theoretical, and modeling evidence supports tapering in the downrange direction of projectile motion.

Basin outline fitting – geometric model of a basin formed in oblique impact

In addition to the simple tapered ellipse used to fit the basin outlines, we also fit the basin outline with a simple geometrical model based loosely on the physics of tapered basin formation. As discussed above, projectile decapitation, planetary curvature, and the downrange impact of the core of a differentiated projectile may all play roles in the tapering. We here focus on the effect of planetary curvature since it is amenable to a simple analytic model that can be used to define a tapered basin shape for fitting to the observed basin outlines as described below. While this model and its success in matching observed basin shapes may provide some insight into the formation of tapered elongated impact basins, it is intended here primarily for geometric purposes of fitting the shapes of observed basins.

We begin with a simplified version of the geometric model for elliptical basin formation based on the concept of the expansion of the projectile footprint to form the basin[20] (Supplementary Fig. 1). For a projectile of radius r impacting a planet of radius r_p at an impact angle θ at the point of first contact at x=0, the x coordinates of the intersection of the leading and trailing edges of the projectile with the planet are calculated as:

$$x_{\pm} = \pm\frac{-b-(b^2-4ac)^{\frac{1}{2}}}{2a}$$

$$a = tan^2(\theta)+1$$

$$b = \mp 2r\cdot sin(\theta)tan^2(\theta) - 2tan(\theta)\left(r_p + r \pm r\cdot cos(\theta)\right)$$

$$c = r^2 sin^2(\theta)tan^2(\theta) \pm 2r\cdot sin(\theta)tan(\theta)\left(r_p + r \pm r\cdot cos(\theta)\right) + \left(r_p + r \pm r\cdot cos(\theta)\right)^2 - r_p^2$$

From these x-coordinates, the major and minor axes of the projectile footprint as measured along the curving surface of the target planet are:

$$A_p = r_p\left(arcsin\left(\frac{x_+}{r_p}\right) + arcsin\left(\frac{x_-}{r_p}\right)\right)$$

$$B_p = 2r_p arcsin\left(\frac{x_+}{r_p}\right)$$

The basin is next assumed to expand outward from this projectile footprint uniformly in all directions by an amount Δ to yield the major and minor axes of the basin as:

$$A = A_p + 2\Delta$$

$$B = B_p + 2\Delta$$

As in the original work, giant basin-forming impacts are more likely to generate elliptical basins because of the combined effect of the greater elongation of the projectile footprint due to the curvature of the planetary surface, and the decreased expansion Δ of the final basin diameter from the projectile diameter for larger impacts predicted by scaling relations and hydrocode models. In the original formulation[20], only the elongation of the basin was of interest and the model did not account for the tapering of the basin in the downrange direction. Here, the basin is assumed to taper in the downrange direction by scaling the cross-track width by $\sin^{1/3}(\theta')$ where θ' is the local angle between the direction of projectile motion and the surface[28,29,89]. The dependence of crater diameter on impact angle is usually applied to the mean diameter and mean impact angle based on experiments and models[28,29,89], but is here applied to the local impact angle which varies across the basin. In the MCMC, the projectile radius r, the expansion term Δ, and the impact angle at point of first contact θ are varied to achieve the best-fit to the basin outline. This model does not explicitly consider impact velocity, which is related to the expansion of the projectile footprint to form the basin. For basins at the limit of projectile decapitation, this model makes the unphysical prediction of the downrange end tapering to a point because this model does not take into account the downrange scouring that occurs when projectile motion is parallel to the surface at this point. While this is not observed in nature, basins formed in decapitating impacts do exhibit distinctive morphologies[30], as evidenced by Sputnik basin[35].

This geometrical model is simplistic but captures one aspect of the basic phenomena responsible for the elongation and tapering of impact basins and some craters and provides a simple analytic expression relating basin shape to impact parameters that can be called from a Monte Carlo model to fit the basin outlines. Although a full hydrocode model of basin formation[3,24,36,37]

would be preferable, such models are computationally intensive and not feasible within a Monte Carlo framework and not yet capable of fitting the observed range of basin shapes.

Although this geometrical model is framed in terms of the projectile radius and impact angle, those values should not be interpreted as robust estimates of either. Rather, within the context of this geometrical model, which is loosely based on the physics of the formation of an elongated tapered impact basin in an oblique impact, these are the parameters that provide the best-fit to the basin outline. This model does serve to demonstrate that a simple understanding of the effect of impact angle on crater expansion can predict basin shapes that are generally consistent with those that are observed (Supplementary Fig. 2; Supplementary Table 1). For a number of the basins, including SPA, this model predicts greater tapering of the best-fit outline than the simple tapered ellipse. As with the previous model, different outline picks yield different tapering values. This result highlights the fact that the particular value of the tapering of the basin shape depends on both the choice of outline and the choice of shape and its parameterization in fitting that outline.

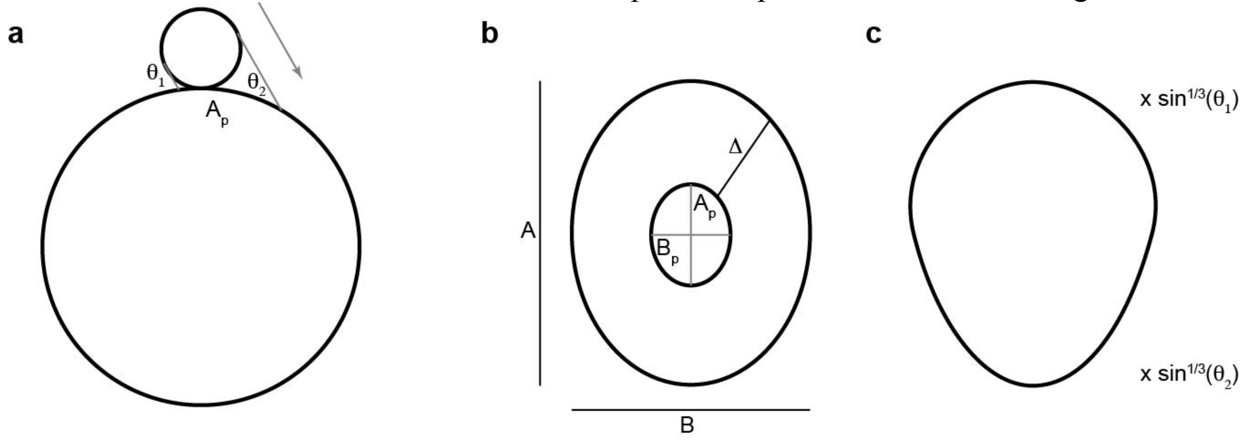

Supplementary Figure 1. Schematic representation of the geometric model of tapered basin formation. **A** A projectile striking a planetary surface at an oblique angle has different effective impact angles at the uprange (θ_1) and downrange (θ_2) extremes of the projectile footprint. **B** An elongated basin forms when the elliptical projectile footprint of dimensions A_p and B_p expands by a uniform amount Δ in each direction, producing a basin of dimensions A and B. **C** The cross-track width of the final basin is scaled by the cube root of the sine of the local impact angle, resulting in a basin outline that tapers in the downrange direction.

Supplementary Figure 2. (next page) Fits to the basin outlines using the geometrical model based on an oblique impact onto a curved planetary surface. Panels **a-f** are as in Figure 1, and panels **g-n** are as in Supp. Fig. 1. Shown are the preferred fits for SPA from gravity overlain on Bouguer gravity (**a**) and gravity gradient (**b**); the preferred fit for SPA overlain on topography (**c**); fit for Hellas (**d**), Smythii (**e**) and Sputnik (**f**) basins from topography; two alternate outline choices for SPA from gravity (**g,h**), an alternate fit for SPA from topography (**i**); fits based on Bouguer gravity for Hellas (**j**), Crisium (**k**), and Utopia (**l**); and fits for Smythii using topography (**m**) and gravity (**n**). Models approaching or exceeding the projectile decapitation threshold result in a pointed downrange end because the effects of the decapitated portion of the projectile are not accounted for. Each panel is in a basin-centered polar projection with the inferred down-range direction to the bottom.

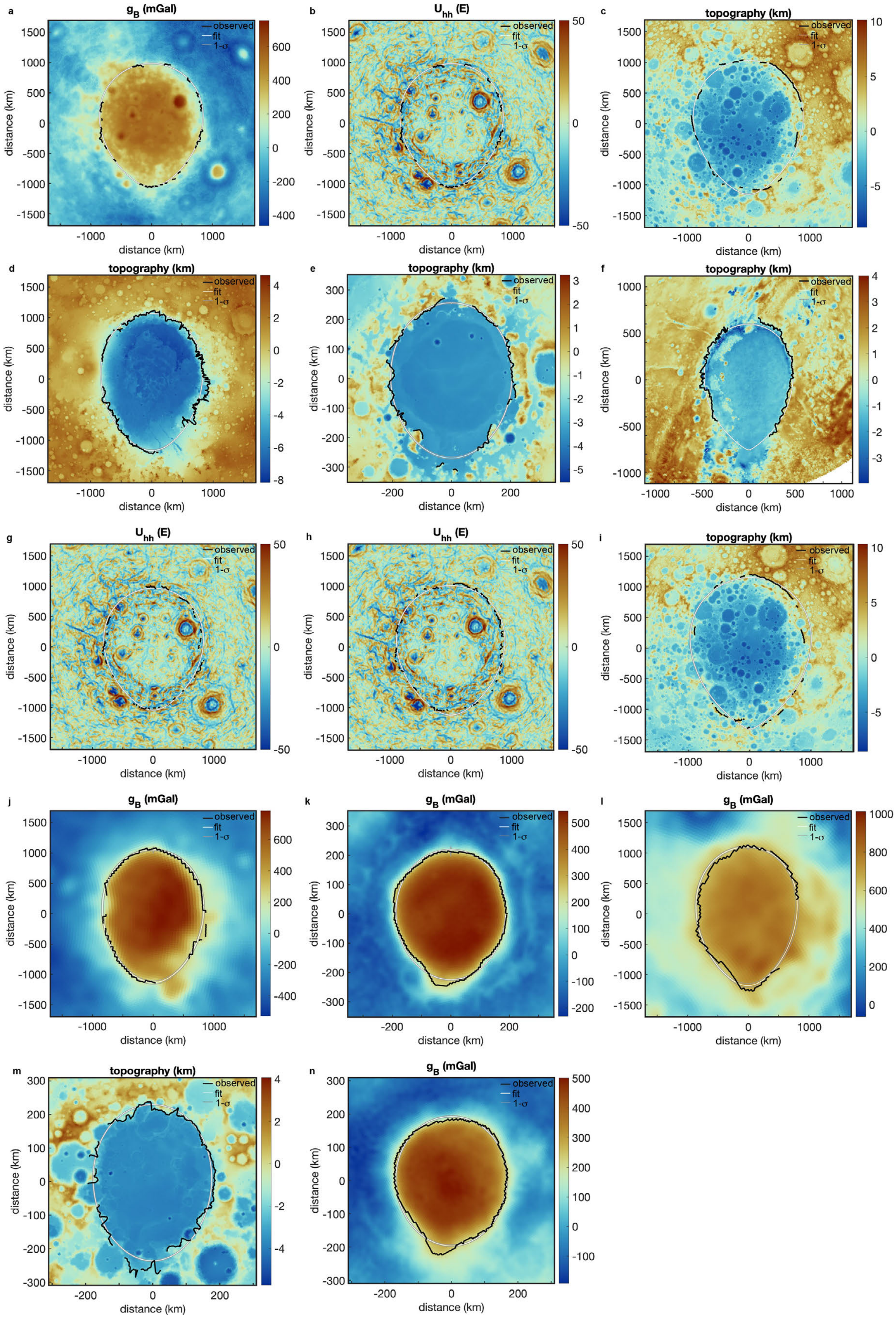

a
g_B (mGal)
b
U_{hh} (E)
c
topography (km)
d
topography (km)
e
topography (km)
f
topography (km)
g
U_{hh} (E)
h
U_{hh} (E)
i
topography (km)
j
g_B (mGal)
k
g_B (mGal)
l
g_B (mGal)
m
topography (km)
n
g_B (mGal)
observed
fit
1-σ
distance (km)

Supplementary Table 1. Monte Carlo model results for the geometrical model of an oblique impact onto a curved planetary surface. Mean and 1-σ range of key parameters are given from the Monte Carlo model for the outline fits.

Basin	outline data	$r_{projectile}$ (km)	θ	f (tapering)
SPA	U_{hh}, g_B, 1	198±10	52±2°	0.25±0.02
	U_{hh}, g_B, 2	194±5	51±2°	0.28±0.02
	U_{hh}, g_B, 3	212±7	54±2°	0.28±0.02
	z,1	214±7	52±2°	0.31±0.02
	z, 2	234±8	50±2°	0.38±0.02
Hellas	z	117±33.	36±5°	0.22±0.03
	g_B	152±32	41±5°	0.19±0.02
Utopia	g_B	121±33	35±5°	0.23±0.03
Sputnik	z	111±3	42±4°	0.37±0.04
Crisium	z	30±6	39±4°	0.09±0.01
Crisium	g_B	27±6	46±4°	0.06±0.01
Smythii	z	24±7	35±3°	0.09±0.01
Smythii	g_B	27±6	48±3°	0.05±0.01

Additional discussion of ejecta composition.

The surface composition across the entire Moon has been modified by the ejecta of basins and craters of all sizes and ages. Portions of the SPA melt sheet on the basin floor show clear evidence for compositional redistribution and overprinting by volcanism and recent impacts[11]. However, the SPA rim region is uniformly covered in craters and basins and there is no reason to expect that the effects of ejecta from later impacts would cause the striking difference in Th concentration between the northeastern and southwestern ejecta blankets of SPA. In the northeastern portion of the floor of SPA, the ejecta of the Apollo basin has clearly affected the surface composition, leading to lower concentrations of Th within the outer ring of Apollo in comparison the nearby portions of the SPA melt sheet on the basin floor[11]. However, the effect of Apollo ejecta on surface composition does not extend far beyond the outer ring at a distance of 250 km from the basin center, since the thinner distal ejecta would have been mixed into the underlying crust by later impacts. The low-Th portion of the SPA ejecta blanket extends >1000 km from the center of Apollo. Moreover, while the Apollo ejecta has a lower Th concentration than the SPA melt sheet in the basin center, it has a higher Th concentration than found in the low-Th SPA ejecta and should cause an increase rather than decrease in Th concentration there. Thus, ejecta from Apollo cannot explain the lack of Th signature in the northeastern SPA ejecta.

The southern portions of the low-Th SPA ejecta are closer to the Mendel-Rydberg basin, but there is no clear compositional anomaly associated with this ancient basin. In between, much of the low-Th ejecta of SPA lies at distances of ~600 km from either basin, far from the influence of their ejecta. Models of the combined thickness of the ejecta of all lunar basins[90] predict that the northeast ejecta blanket of SPA lies in a region with the lowest contribution from non-SPA ejecta of anywhere on the Moon, with total thicknesses of 125-275 m. The substantial vertical and lateral mixing of the megaregolith in the time during and since the basin-forming era would have mixed this thin ejecta with the underlying thicker SPA ejecta[91]. Similarly, there is no obvious correlation between the distribution of the Th-rich southwestern ejecta blanket and other craters in the region. While basin ejecta has the potential to cover the surface in places, the SPA ejecta would have

reached thickness up to ~20 km in the vicinity of the rim[36], and would be continually re-exposed and mixed into the surface materials by the numerous smaller post-SPA impacts[91]. Thus, the dominant surface compositions in the northeastern and southwestern SPA ejecta blanket are taken to be representative of the SPA ejecta.

Both Th and Ti have distinctive signatures in the ejecta and melt sheet of SPA. It has commonly been assumed that the KREEP-rich dregs of the magma ocean and the over-dense Ti-rich cumulates were coupled during early lunar evolution and migrated to the nearside together[70], but the inclusion of KREEP with the dense Ti-rich cumulates is not required in those models and this assumption is neither clearly supported by observations nor necessarily expected to be the case. The spatial distribution of Th on the nearside is heavily affected by the distribution of Th-rich ejecta from the Imbrium impact[46]. The lack of a notable Ti signature in that ejecta suggests that the KREEP-rich reservoir in the target was not similarly enriched in Ti. Rather, the nearside distribution of Ti is dominantly a result of Ti-rich mare basalts. These Ti-rich basalts were likely the result of partial melt of Ti-rich cumulates in the mantle, possibly associated with a buoyant upwelling of warmed materials[92,93] (which may have required the presence of some KREEP-rich materials in those cumulates). Thus, the vertical and lateral distribution of Th and Ti at and near the surface of the nearside are not necessarily coupled.

The details of the crystallization of the late stages of the magma ocean are not well constrained, with some studies assuming the final liquids crystallized *en masse* at some arbitrary point[94]. However, recent models[5] predict the late stages of magma ocean crystallization to have been exceedingly slow, extending the total crystallization time to >100 Myr. At the same time, the density contrast of the late-stage Ti-rich cumulates relative to the co-existing magma ocean would have been much greater than the density contrast between the cumulates and magma at any other time[42]. Given the long crystallization timescale and strong density contrast, efficient separation of the crystals and liquid may be expected[75]. It must also be noted that crystallization of Ti-rich phases begins relatively early (~90 PCS), while Th concentrations do not reach levels thought to be representative of the final KREEP reservoir until much later (>99 PCS; see Fig. 4). Thus, while both Ti-rich cumulates and KREEP-rich liquids are late products of magma ocean crystallization and both are ultimately concentrated on the nearside, they should not be treated as a single entity in space and time. As with Imbrium, the observation of Th-rich but Ti-poor ejecta around SPA reinforces this view.

The Th and Ti concentrations in the basin floor, east ejecta, and highlands can easily be fit with a linear relationship at the 2-σ confidence level (p=0.03), though the fit is not meaningful given the similarity between the east ejecta and the highlands (Fig. 4a). However, if the west ejecta is included a linear fit is no longer significant (p=0.13), supporting the interpretation that three compositional reservoirs are required as discussed in the main text. The lack of a strong Ti signature in either the east or west ejecta blankets of SPA but its presence in the central melt sheet of the basin suggests that either the impact did not excavate as deeply as predicted in some models[2,3] or that dense Ti-bearing cumulates in the lunar farside may have mixed into the underlying cumulate mantle as they formed[95] rather than later as a discrete layer[51]. In the latter case, gravitational evidence for remnants of sinking slabs of dense Ti-rich material on the nearside[52] would indicate that the mode of mantle overturn may have differed between the near and far sides of the Moon, perhaps related to the different temperature and pressure conditions at the top of the mantle[96].

Additional discussion of crustal thickness patterns

As discussed in the main text, the steep crustal thickness gradient in the northern basin rim and gentler gradient in the southern rim of SPA supports a southward impact trajectory. This difference in crustal thickness gradient is observed in crustal thickness profiles when the global long-wavelength patterns of crustal thickness have been removed (Fig. 3; removing spherical harmonic degrees 1 and 2) as well as in profiles without filtering out the long wavelengths (Supplementary Fig. 3a). Elongated basins exhibit a more gradual transition in topography and crustal thickness at the downrange end than the uprange end. In some cases, the more gradual transition is limited to a narrow range of azimuths in the downrange direction, giving the appearance of a narrow downrange "tail" in topography and Bouguer gravity as seen at Crisium, Smythii, Utopia, and Sputnik (Supplementary Fig. 2). This narrow downrange transition zone may be related to the decapitation of the projectile or the downrange effects of the core of the differentiated impactor. Similarly, for SPA the more gradual transition in topography and crustal thickness is also focused narrowly around the downrange end of the basin, with steeper transitions common at other azimuths.

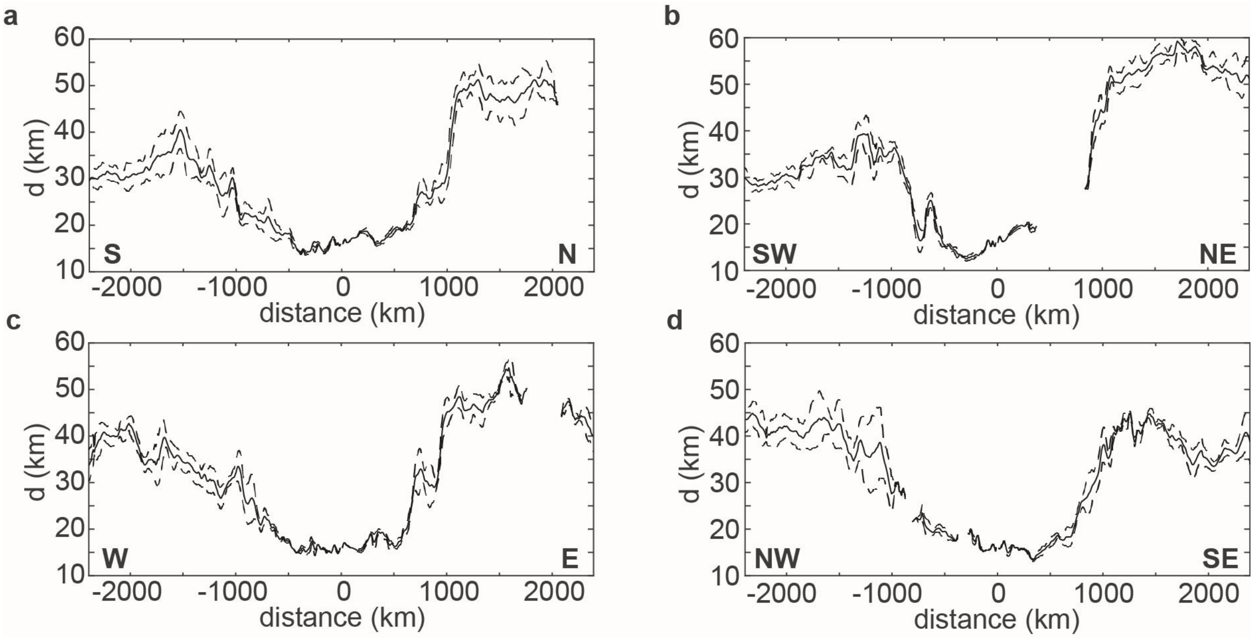

Supplementary Figure 3. Crustal thickness profiles across SPA at 45° intervals. Profiles are taking from a crustal thickness model[65] without removing the long wavelengths, averaged over 15° of azimuth. Younger basins were masked out prior to averaging resulting in gaps in some profiles.

A more gradual transition is also found in the northwestern quadrant of the basin, breaking the bilateral symmetry (Fig. 3; Supplementary Fig. 3d). This quadrant corresponds with a local area of particularly thin crust outside the basin rim and may have been underlain by a thicker magma ocean layer at the time of impact, possibly leading to greater post-impact collapse and relaxation. The locally thinner crust northwest of the basin may also explain the higher Th concentrations in the area. A west-east profile across the basin (Supplementary Fig. 3c) crosses a set of large linear gravity anomalies west of the basin likely associated with later intrusive activity[31], leading to the appearance of a thinner crust and a more gradual transition. In contrast, there is no evidence that the uprange or downrange crustal thickness gradient has been affected by

either pre- or post-impact processes, and the direction of impact is the likely explanation for the uprange/downrange asymmetry.

Alternative scenarios: impact trajectory

A northward trajectory for the SPA impact was initially based on the suggestion that SPA ejecta may contribute to the crustal and topographic asymmetry as supported by the volume equivalence of the crustal deficit in the basin center and crustal excess of the farside highlands[21]. However, subsequent hydrocode models of the basin-forming impact have not generated crustal thickening downrange of the basin consistent with the observed pattern of crustal thickening in the farside highlands. Models predict crustal thickening focused primarily downrange[3] and cross-range[36,37] of the basin and symmetric about the long-axis of the basin. In contrast, the crustal thickening of the farside highlands is primarily to the east of the long axis of the basin (Supplementary Fig. 4a; not removing spherical harmonic degrees 1 and 2). While crustal-thickness based models of basin ejecta can be constructed that are quasi-symmetric about the basin long axis, removal of this ejecta would leave behind a substantial crustal thickness asymmetry[22,73]. Since the crustal thickness asymmetry cannot be fully explained by SPA ejecta, and since the crustal thickness excess of the farside highlands does not match the expectation for an ejecta blanket focused downrange and symmetrically about the long axis, there is no obvious reason to use the observed crustal thickness distribution as evidence supporting a northward impact trajectory. No such prominent crustal thickening or topographic excess is seen around other basins, as exemplified by Hellas on Mars (Supplementary Fig. 4b). However, the observed crustal thickness alone does not explicitly argue against a northward impact trajectory, nor can we exclude the possibility that SPA ejecta contributes to the farside highlands crust. Interestingly, the ejecta from an impact on a southward trajectory as proposed here may increase the magnitude of the global asymmetry if removed from the present-day topography and crustal thickness.

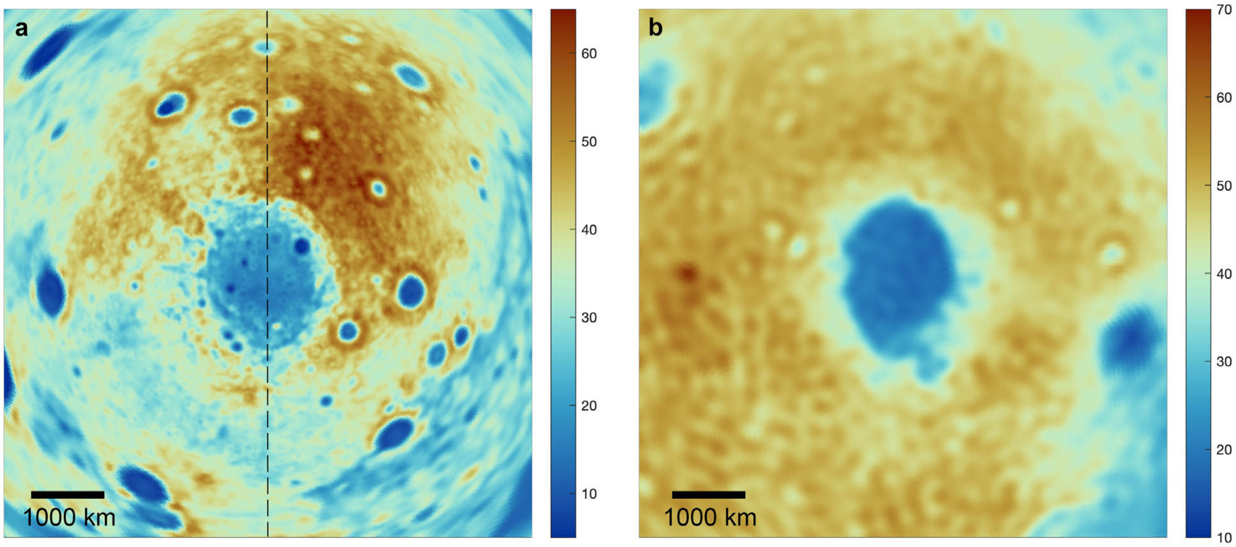

Supplementary Figure 4. Comparison of the crustal thickness distribution around SPA and Hellas impact basins. Basins are shown in basin-centered radial projections (extending to greater distances and not filtering out the long wavelength degree 1 and 2 variations for SPA as was done in Figure 2). Both basins are shown at the same horizontal scale and color stretch, with the long-axis of each basin oriented vertically. The farside highlands crustal thickening features prominently to the top right of SPA and is clearly offset relative to the symmetry axis (dashed line).

Arguably the strongest evidence for a northward impact trajectory is the interpretation that the magnetic anomalies in the farside are dominantly found in the northern rim region of the basin, where projectile mantle and core are predicted to be intermixed with the crust for a northward impact trajectory[24]. These magnetic anomalies are concentrated in the transition region between the thick crust outside the basin and the thin crust of the basin floor where projectile core material is predicted. However, the magnetic anomalies also extend ~1000 km beyond the basin rim, where those models predict ejecta dominated by projectile mantle with a low magnetic susceptibility for a differentiated projectile. We also note a concentration of strong magnetic anomalies centered at 17°S 237°E, ~700 km from the basin rim in the direction orthogonal to the long axis of the basin, which are of comparable scale and magnitude to those at the SPA rim. Basin ejecta should be relatively thin in this region and is not expected to contain any projectile core material. Other smaller magnetic anomalies are found elsewhere on the far and near sides. If SPA ejecta is invoked to explain the magnetic anomalies in the basin rim region, then some other mechanism is required to explain these other magnetic anomalies such as the focusing of magnetic field lines antipodal to nearside basins[25]. Although we cannot discount the possibility that magnetic anomalies in the vicinity of SPA represent basin ejecta and support a northward impact trajectory, this is an indirect constraint that is somewhat weakened by the need for other independent explanations for other anomalies. Alternatively, a downrange focusing of either projectile core material or the thermal effects of the impact may explain the deep mass excess in the southern part of the basin[19], consistent with a southward impact. We consider the basin shape, in both outline and cross-section, to be a more direct constraint on projectile motion.

Recent work mapped the distribution of topographically distinct massifs around the basin and fit them with elliptical ring stuctures[97]. The results showed a largely circular pattern interpreted as suggesting a near-vertical impact, in conflict with this study as well as previous interpretations of the shape of the basin from topography, crustal thickness, and remote sensing data[18–20]. The massifs in the new mapping are found to lie close to the topographic rim in the eastern half of the basin and begin at the rim but extend outward to greater distances in the western half, such that the inner edge of the distribution of massifs follows an elliptical shape similar to previous mappings of the rim while the outer edge of the distribution is more circular. This distribution of massifs closely resembles the distribution of large-magnitude small-scale gravity anomalies interpreted as the gravitational signature of mantle-bearing ejecta that is asymmetrically distributed due to the effects of the impact trajectory and variations in pre-impact crustal thickness across the basin[39,98], and the massifs themselves superficially resemble massifs east of the Hellas basin on Mars interpreted as ejecta[33]. This similarity suggests that the massifs represent ejecta rather than basin rim or ring segments, and thus their distribution does not support a non-oblique impact.

Alternative scenarios: Compositional stratigraphy

Previous studies inferred a compositional stratigraphy based on the high concentrations of Th and Ti throughout the basin floor that were interpreted to be related to the basin ejecta and/or impact melt[11,12,38]. Our work is broadly consistent with and reinforces those interpretations, while shedding new light on the origin of the compositional anomalies and the implications for the impact and early lunar evolution. In the previously proposed compositional stratigraphy at the time of impact[11], the upper mantle excavated by the basin consisted of late-stage cumulates that were uniformly enriched in Th and Ti. This Th-rich mantle material was interpreted to be exposed in the collapsed ejecta blanket in a crescent interior to the rim in the northwest portion of the basin

with Th concentrations of 3–4.5 ppm, which would favor a northward projectile trajectory. The somewhat lower concentrations of Th (2–3 ppm) in the central parts of the basin were interpreted as being related to more deeply generated impact melt sourced from deeper levels of cumulates less enriched in Th. These observations and interpretations provided important evidence for a compositionally stratified lunar mantle at the time of the impact[11] and are broadly consistent with the model advanced in this study. However, our work differs in the interpretation of the subsurface stratigraphy and the distribution of impact melt and ejecta on the basin floor based on geophysical and geochemical evidence.

Basin ejecta and melt sheet distribution. Both impact melt and ejecta is expected interior to the basin rim[11], but the true extent of each unit is uncertain. Three-dimensional impact simulations end with an annulus of ejecta-thickened crust surrounding the basin, which is inferred to collapse into the basin interior[3,36,37]. Two-dimensional simulations of the impact (limited to vertical impacts) predict that this thick ejecta deposit will spread inward to either partially[2] or completely[99] cover the basin floor. The thickest parts of the ejecta blanket should contain a mixture of crust and mantle[3,36]. Recent geophysical analyses have revealed an annulus of short wavelength and high magnitude gravity anomalies around the basin (Supplementary Fig. 5), interpreted as impact ejecta composed of a mixture of high-density material derived from the mantle and low density material derived from the crust[39,94]. This annulus begins interior to the basin rim and extends out over the surroundings. Interior to this annulus lies a central zone of weak gravity anomalies and more uniform density interpreted as the crystallized melt sheet[39,98]. This inner zone could contain a mixture of deeply sourced impact melt, melt from the ejecta that flowed into the basin interior, remnant magma ocean liquids from the surroundings, and additional solid ejecta that became mixed and homogenized with the melt sheet.

The crescent containing the highest Th concentrations (>3 ppm) in the northwest portion of the basin floor is found almost entirely within the central zone of weak gravity anomalies, indicating that it is associated with the central melt sheet rather than ejecta (Supplementary Fig. 5). Variations in Th concentration and mineralogy within this inner zone previously interpreted as the contrast between impact melt and ejecta may instead be related to volcanic resurfacing by mare and cryptomare in the central zone[100–102]. The Th concentrations in the ejecta within the rim region immediately adjacent to the central melt sheet may have been affected by the extreme mobility of material within the basin during the collapse of the transient cavity, as well as by the later mixing and redistribution of material by impacts[11] and the low resolution of the GRS instrument. In contrast to the basin floor and adjacent rim, Th-rich material in the west ejecta blanket beyond the rim must be derived from basin ejecta, allowing for a more direct constraint on the ejecta composition.

Deep compositional stratigraphy. An alternative view is that the impact excavated into a thicker upper mantle layer uniformly enriched in Th[11]. Although not suggested in the previous study, one might interpret the difference in Th concentration between the southwest and northeast ejecta blankets as consistent with this scenario of a mantle uniformly enriched in Th but that was only excavated in the southwestern part of the basin because of the thinner crust. However, as shown below, this scenario cannot explain either the required concentrations of Th or the distribution of Th-rich ejecta around the basin.

The Th concentration in the late-stage magma ocean and any materials derived from it is inversely proportional to the thickness of the layer due to the partitioning of Th into the melt as discussed in the main text. The observed Th concentrations within the floor of SPA of up to 4.5 ppm are only obtained at ~98.8 PCS, which would correspond to a globally uniform layer only 6–

7 km thick for an initial magma ocean depth of 1000–1337 km. For this layer to exist globally just below the base of the crust would require 10's of km of relief and strong departures from hydrostatic equilibrium. This situation is not compatible with an isostatically floating crust forming above the late-stage magma ocean, which would instead result in lateral variations in thickness of the magma ocean and resulting cumulates, leading to a globally discontinuous layer at this late stage as shown above. Thus, to attain the concentrations found on the basin floor as part of the collapsed basin ejecta would require the scenario proposed here of a thin and discontinuous late-stage magma ocean. However, this thin layer would also comprise a small fraction of the ejecta for expected excavation depths exceeding 100 km (refs. [3,36]), and would lead to lower concentrations in the ejecta than found on the basin floor, as is seen in the west ejecta blanket. For a thicker layer of Th-rich late-stage cumulates in the upper mantle that could be globally distributed and would dominate the excavated material both outside and within the basin[11], the Th concentration would be much lower. For a 50-km-thick layer (91 PCS), the Th concentration would be 0.9 ppm, much lower than observed in both the high-Th material on the floor of SPA and in the southwest ejecta blanket.

Asymmetric excavation of a thick upper mantle layer enriched in Th due to the variations in crustal thickness also cannot explain the observed compositional asymmetry in the ejecta blanket. Models predict excavation of crust and mantle throughout the basin, with excavation depths of up to 100 km (refs. [3,36]). More significantly, as discussed above, gravity data reveals the distribution of heterogeneous mantle-bearing ejecta around the basin, which is found in both the east and west ejecta blankets. The region of mantle-bearing ejecta to the west exhibits strong gravity anomalies and elevated Th concentrations (~2 ppm) consistent with excavation of both dense mantle material and late-stage magma ocean liquids, while the region of mantle-bearing ejecta to the east exhibits strong gravity anomalies and lower Th concentrations consistent with excavation of mantle material only (Supplementary Fig. 5). Thus, excavation of Th-poor mantle and a separate Th-rich layer is required.

Although this study focuses on the Th-rich ejecta blanket beyond the rim, the origin of the high Th concentrations on the basin floor must also be explained. If the excavated Th-rich material was a thin and discontinuous layer of late-stage magma ocean liquids as suggested here, it should have been fully excavated from beneath the basin and not contribute significantly to the central impact melt sheet. Instead, the impact melt should be derived primarily from the upper ~150 km of mantle cumulates[2,11], which are generally expected to be poor in Th due to its partitioning into the magma ocean during fractional crystallization. Low concentrations of Th are expected in these cumulates, largely as a result of the small amount of melt trapped between the crystals during growth of the cumulate pile. Additional Th may have been present in the melt sheet as a result of the incorporation of impact ejecta and magma ocean liquids. However, this melt pool itself would undergo fractional crystallization, with Th and other incompatible elements concentrated in the latest liquids and the solids derived from them. The 50-km-thick melt sheet could have differentiated to generate the 12–20 km thick crust and new mantle beneath the basin floor[12,38]. For such a thick melt sheet, the final 1 km of melt and the solids derived from them would have a 50-fold increase in Th concentration relative to the original mantle cumulates melted by the impact. Thus, for example, if the late-stage lunar magma ocean cumulates trapped 2% melt within their interstices, the final kilometer of solids generated from the SPA melt sheet would have a Th concentration comparable to the late-stage magma ocean liquids. Higher concentrations could be generated from even later stage liquids in the melt sheet. The lateral variations in Th concentration on the basin floor may be attributed to the original distribution of the final solids formed, to vertical

and lateral redistribution of material by impacts and volcanic resurfacing[11], and to the possible overturn of the cumulates in the crystallized melt sheet[12].

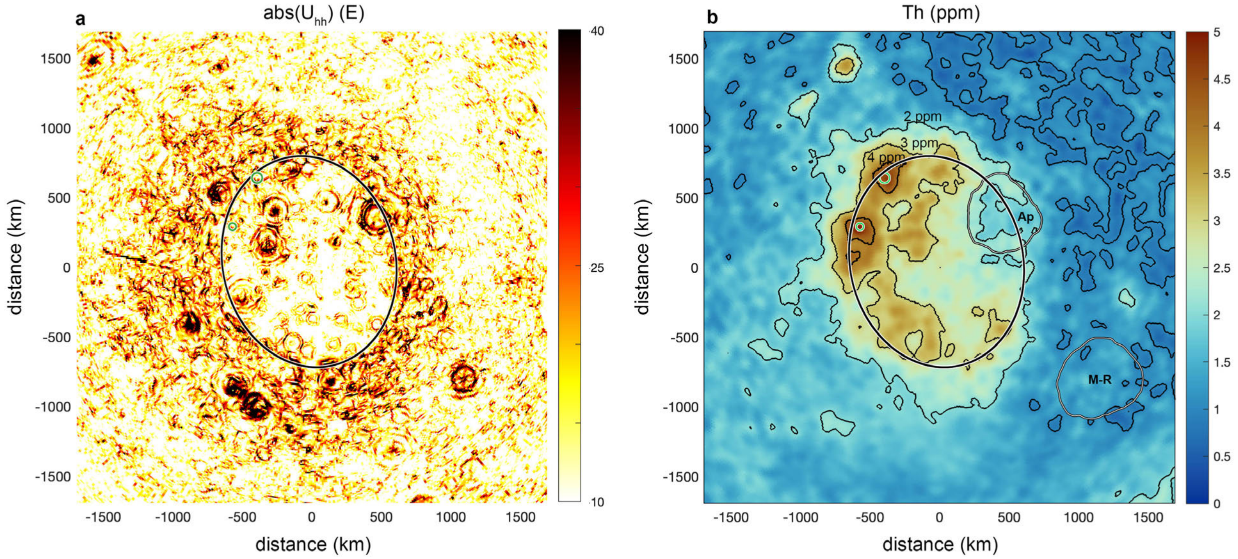

Supplementary Figure 5. Comparison of the magnitude of the gravity gradients with the thorium distribution. Gravity gradients (**a**) were calculated as the maximum amplitude eigenvalue of the horizontal second derivatives of potential evaluated at the surface[31,61] using GRAIL gravity data. A central ellipse of weak gravity anomalies (outlined in both panels) is interpreted as the uniform density crystallized melt sheet, which is surrounded by an annulus of large-magnitude short-wavelength gravity anomalies interpreted as mantle-bearing ejecta[39]. The crescent of high (>3 ppm) Th concentrations (**b**) lies almost entirely within the central melt sheet unit, with excursions into the ejecta annulus associated with dispersal by impact craters[11] (gray circles). The locations of the Apollo (Ap) and Mendel-Rydberg (M-R) basin outer rings are shown in **b**. The projection is as in Fig. 1a.

Supplementary References

80. Schultz, P. H. & Gault, D. E. Decapitated impactors in the laboratory and on the planets. *Lunar Planet. Sci. Conf.* **21**, abstract 1099 (1990).
81. Bottke, W. F., Love, S. G., Tytell, D. & Glotch, T. Interpreting the elliptical crater populations on Mars, Venus, and the Moon. *Icarus* **145**, 108–121 (2000).
82. Collins, G. S., Elbeshausen, D., Davison, T. M., Robbins, S. J. & Hynek, B. M. The size-frequency distribution of elliptical impact craters. *Earth Planet. Sci. Lett.* **310**, 1–8 (2011).
83. Schultz, P. H. & Lutz-Garihan, A. B. Grazing impacts on Mars: A record of lost satellites. *J. Geophys. Res. Solid Earth* **87**, A84–A96 (1982).
84. Andrews-Hanna, J. C., Zuber, M. T. & Banerdt, W. B. The Borealis basin and the origin of the martian crustal dichotomy. *Nature* **453**, 1212–1215 (2008).
85. Phillips, M. S. *et al.* Extensive and ancient feldspathic crust detected across north Hellas rim, Mars: Possible implications for primary crust formation. *Geology* **50**, 1182–1186 (2022).
86. Neumann, G. A. *et al.* Crustal structure of Mars from gravity and topography. *J. Geophys. Res.* **109**, E08002, doi:10.1029/2004JE002262 (2004).
87. Schenk, P. M. *et al.* Basins, fractures and volcanoes: Global cartography and topography of Pluto from New Horizons. *Icarus* **314**, 400–433 (2018).
88. Potter, R. W. K., Kring, D. A., Collins, G. S., Kiefer, W. S. & McGovern, P. J. Estimating transient crater size using the crustal annular bulge: Insights from numerical modeling of lunar basin-scale impacts. *Geophys. Res. Lett.* **39**, L18203 (2012).
89. Davison, T. M. & Collins, G. S. Complex crater formation by oblique impacts on the Earth and Moon. *Geophys. Res. Lett.* **49**, e2022GL101117 (2022).
90. Petro, N. E. & Pieters, C. M. The lunar-wide effects of basin ejecta distribution on the early megaregolith. *Meteorit. Planet. Sci.* **1529**, 1517–1529 (2008).
91. Liu, T., Michael, G., Engelmann, J., Wünnemann, K. & Oberst, J. Regolith mixing by impacts: Lateral diffusion of basin melt. *Icarus* **321**, 691–704 (2019).
92. Zhong, S., Parmentier, E. M. & Zuber, M. T. A dynamic origin for the global asymmetry of lunar mare basalts. *Earth Planet. Sci. Lett.* **177**, 131–140 (2000).
93. Zhang, N., Parmentier, E. M. & Liang, Y. A 3-D numerical study of the thermal evolution of the Moon after cumulate mantle overturn: The importance of rheology and core solidification. *J. Geophys. Res. Planets* **118**, 1789–1804 (2013).
94. Elkins-Tanton, L. T., Burgess, S. & Yin, Q. The lunar magma ocean: Reconciling the solidification process with lunar petrology and geochronology. *Earth Planet. Sci. Lett.* **304**, 326–336 (2011).
95. Maurice, M., Tosi, N. & Hüttig, C. Small-scale overturn of high-Ti cumulates promoted by the long lifetime of the lunar magma ocean. *J. Geophys. Res. Planets* **129**, e2023JE008060 (2024).
96. Laneuville, M., Taylor, J. & Wieczorek, M. A. Distribution of radioactive heat sources and thermal history of the Moon. *J. Geophys. Res. Planets* **123**, 3144–3166 https://doi.org/10.1029/2018JE005742 (2018).
97. Bernhardt, H. *et al.* Numeric ring-reconstructions based on massifs favor a non-oblique south pole-Aitken-forming impact event. *Earth Planet. Sci. Lett.* **650**, 119123 (2025).
98. Gowman, G. *et al.* The lunar mantle below the South Pole-Aitken basin rim. *Lunar Planet. Sci. Conf.* **56**, abstract 1563 (2025).

99. Trowbridge, A. J., Johnson, B. C., Freed, A. M. & Melosh, H. J. Why the lunar South Pole-Aitken Basin is not a mascon. *Icarus* **352**, 113995 (2020).
100. Wang, X. *et al.* Lunar farside South Pole-Aitken basin interior: Evidence for more extensive central cryptomaria in the South Pole-Aitken Compositional Anomaly (SPACA). *J. Geophys. Res. Planets* **129**, e2023JE008176 (2024).
101. Moriarty III, D. P. & Pieters, C. M. The Character of South Pole-Aitken Basin: Patterns of surface and subsurface composition. *J. Geophys. Res. Planets* **123**, 729–747, https://doi.org/10.1002/2017JE005364 (2018).
102. Ivanov, M. A. *et al.* Geologic History of the northern portion of the South Pole-Aitken Basin on the Moon. *J. Geophys. Res. Planets* **123**, 2585–2612 https://doi.org/10.1029/2018JE005590 (2018).
